# GIS for empirical research design: An illustration with georeferenced point data

**DOI:** 10.1371/journal.pone.0212316

**Published:** 2019-03-04

**Authors:** Katsuo Kogure, Yoshito Takasaki

**Affiliations:** 1 Center for Southeast Asian Studies, Kyoto University, Kyoto, Japan; 2 Graduate School of Economics, University of Tokyo, Tokyo, Japan; Brown University, UNITED STATES

## Abstract

This paper demonstrates how Geographic Information Systems (GIS) can be utilized to study the effects of spatial phenomena. Since experimental designs such as Randomized Controlled Trials are generally not feasible for spatial problems, researchers need to rely on quasi-experimental approaches using observational data. We provide a regression-based framework of the key procedures for GIS-based empirical research design using georeferenced point data for both spatial events of interest and subjects exposed to the events. We illustrate its utility and implementation through a case study on the impacts of the Cambodian genocide under the Pol Pot regime on post-conflict education.

## Introduction

With the growing availability of spatial data from Global Positioning Systems (GPS) and remote sensing, Geographic Information Systems (GIS)—computer systems designed to gather, store, manage, display, and analyze spatial data—have become an important tool in geographical, environmental, health, and social science research [1, 2]. Although GIS has been used to visualize, process, create, and analyze spatial data across disciplines, its full potential has yet to be realized [3, 4]. This paper demonstrates how GIS can help in designing empirical research to study the effects of spatial phenomena. Although investigating causal questions is one of the most important research themes across disciplines [5], it has received limited attention in spatial contexts [6, 7]. The gold standard for answering causal questions is Randomized Controlled Trials, which can identify the causal effects of interest through random variation in treatment variables generated by researchers. In spatial contexts, this experimental approach is generally not feasible, so researchers need to rely on a quasi-experimental approach using observational data to approximate an experimental study [8].

Spatial data can be classified into three types: (1) geostatistical data collected over a continuous spatial domain (e.g., temperature), (2) area/lattice data collected on a regular or irregular lattice with well-defined boundaries (e.g., population density in an administrative area), and (3) point pattern data consisting of the observed locations of events/objects of interest (e.g., schools) [9]. Focusing on point pattern data, we consider the occurrence of particular ‘events’ as ‘treatments’ in the literature of causal analysis. This way of formulating an empirical question is a new perspective in the literature that enables empirical researchers to connect spatial statistics with causal analysis.

Our general goal is to evaluate the impacts of the occurrence of spatial events (treatments) during a specific period on local subjects. We consider the following situation/setting where researchers can use GIS to design credible and transparent empirical research. First, the impacts of spatial events are limited to the surrounding local areas. Such events may include the placement of local public goods (e.g., schools, health facilities, clean water facilities) and the occurrence of environmental, epidemiological, political, social, and cultural events (e.g., point source pollution, contagious disease, local conflicts, local beliefs/norms). Second, point pattern data of both events of interest and subjects exposed to the events are available. Although this presumption may not be satisfied in many cases at the moment, more and more household and field surveys have collected georeferenced information using a smartphone or tablet with an integrated GPS chip-set [10]. Spatial point data will become even more widely available in the near future, despite continuing privacy concerns [11]. Regarding the two sets of spatial point data, we assume that while the data-generating process of events follows some form of stochastic mechanism, the locations of subjects are fixed: Local residents do not move/relocate in response to the spatial events of interest (or the migration patterns of subjects are similar around the locations of events if migration is prevalent). Although how reasonable this assumption is depends on empirical contexts, it serves as a benchmark setting.

The key question in estimating the treatment effects in spatial contexts is how to address the threat of unobserved spatial confounding factors. Following the potential outcomes framework [12] for a binary treatment variable, extant studies use covariates and spatial (geographic) information—as a proxy for unobserved spatial confounding factors—to construct matched pairs of treated and control units. Specifically, one study [13] proposes an integer programing method that matches directly on covariates and spatial proximity (distance) and other study [14] proposes distance adjusted propensity score that combines spatial proximity with propensity score.

Following the strategy of exploiting spatial proximity for comparison, we (1) limit a global sample to spatially limited (local) samples using point buffers around the locations of events processed by GIS (spatial clusters) and (2) adjust for unobserved factors that affect or are correlated with both outcomes of interest and treatments and are shared within spatial clusters by controlling for spatial cluster fixed effects in a linear regression model. We further limit the sample to subjects within selected spatial clusters where the locations of events are plausibly exogenous (according to the statistical significance test discussed below). Our approach shares the same objective of adjusting for unobserved spatial confounding factors in the literature.

Our approach follows one of Fisher’s three principles of experimental design, “blocking” (“local control”), with the aim of comparing outcomes among more homogeneous groups, with different levels of treatments within blocks, to reduce omitted variable bias systematically [15]. GIS can flexibly implement this principle in spatial contexts. We do not specifically consider/model spatially correlated residuals addressed in some related literature [16, 17]. Rather, we restrict local samples so that residual spatial variability within small spatial clusters is plausibly assumed to be nonsignificant. Since a linear regression model is a primary workhorse in empirical studies across disciplines, our regression-based approach, which considers both binary and continuous treatment variables, should have significant practical merits.

The reminder of this paper is divided into two sections: We first provide a regression-based framework of the key procedures for GIS-based empirical research design and then implement the framework in a case study on historical political events—the Cambodian genocide.

## A framework of GIS-based empirical research

The key procedures for GIS-based empirical research design consist of three stages: *diagnosis*, *design*, and *analysis*.

### Stage 1: Diagnosis

The diagnosis stage examines how the events of interest occur. Based on theories, findings in the literature, and/or anecdotal evidence, researchers must understand the potential determinants of the locations of the events. Visualizing spatial patterns using GIS can facilitate this process.

### Stage 2: Design

The design stage consists of two main tasks: creating treatment variables and constructing a credible analysis sample to approximate an experimental study.

#### Treatment variables

We consider a general case where exactly how each subject is affected (treated) by the events is unknown (if it is known, it directly informs the creation of treatment variables). We assume that subjects living in closer proximity to the locations of events are more strongly treated by the events: The degree to which subjects are exposed to the events depends on the distance from the locations of events. This assumption should be reasonable for various spatial events. By calculating distance between two spatial points using GIS, we can create distance-based treatment variables. If information about the attributes of points (“marks”) is available, it is possible to make more flexible treatment variables. Treatment variables can be binary, discrete, or continuous.

#### Credible sample

This central task consists of two or three steps of constructing a credible analysis sample from a given global sample which represents the population of interest (global sample). The procedure is the same for binary, discrete, and continuous treatment variables.

#### Step 1—Global sample

The first step assesses the exogeneity of locations of events/treatment variables (binary, discrete, or continuous) for the global sample. As commonly done in empirical studies based on a regression-based framework, we statistically examine the joint significance of observed covariates on treatment variables. Rejecting the exogeneity implies that adjusting for their systematic difference is indispensable. This exogeneity check cannot assess the significance of unobserved factors affecting or correlated with both the outcomes of interest and treatments (“confounders”). If such unobserved confounders exist, the global sample fails to capture the treatment effects of interest: The estimates suffer from omitted variable bias.

#### Step 2—Spatial clusters

The second step deals with this potential omitted variable bias. While instrumental variables methods are conventional, it is often difficult to find valid instrumental variables that are correlated with the locations of events (treatments), but not with the outcome of interest. We employ an alternative approach, blocking, by considering point buffers around the locations of events processed by GIS as neighborhoods, which we call *spatial clusters*, and compare outcomes among subjects, with different levels of exposure to the events, within spatial clusters. Since unobserved confounders (e.g., socioeconomic environments) should be similar within small spatial clusters, omitted variable bias can be reduced systematically. Specifically, for each subject, we create a dummy variable for each spatial cluster that takes the value of 1 if the subject belongs to the spatial cluster and 0 otherwise. If the subject belongs to more than one spatial cluster, corresponding more than one dummy variable takes 1 (an example is given in our case study). Adjusting for these dummy variables (*spatial cluster fixed effects*) in the regression model can eliminate unobserved confounders that are constant within spatial clusters. Prior to the analysis stage, we reassess the exogeneity of the treatment variables for this spatially limited (local) sample, adding spatial cluster fixed effects.

In this second step (and the third step discussed below), the size of spatial clusters is a key auxiliary parameter. Although with smaller cluster size, the number of balanced spatial clusters (described below) becomes larger and socioeconomic characteristics within spatial clusters should be more similar, the number of observations within spatial clusters becomes smaller and the variation in the exposure to the events becomes more limited, thus constraining the creation of treatment variables. This bias-variance trade-off needs to be carefully taken into account by researchers when they choose cluster size. Choosing the optimal size of spatial clusters based on data-driven approaches is beyond the scope of this paper.

#### Step 3—Balanced spatial clusters

If concerns about omitted variable bias still remain in this local sample, the third step is called for. Although omitted variable bias cannot be directly assessed, it may be indirectly assessed through the correlations between the treatment variable and observed covariates (which are relevant according to theories, contextual knowledge, and/or findings in related literature) as well as their joint significance, because unobserved confounders about which researchers are typically concerned are those related to observed confounders [18]: Their significant correlations may imply that some unobserved confounders (that are related to observed confounders) vary within spatial clusters. Then, the estimates based on the local sample may still suffer from omitted variable bias. With information of the location determinants examined in the first diagnosis stage (that affects the occurrence of events), GIS can be utilized to alleviate the remaining concerns about omitted variable bias, as follows. Here we assume that a key binary location determinant is available.

We employ the following procedure for each spatial cluster. First, we additionally create small buffers around the location of the event and classify subjects into the buffers. Second, we create a crosstabulation on the distribution of the key binary location determinant across the buffers. Lastly, we examine the homogeneity of the distribution based on Fisher’s exact test, a statistical significance test for independence between two categorical variables [19]. Based on the results, we further limit the subjects to those within selected spatial clusters with the homogeneous distribution of the location determinant, which we call *balanced spatial clusters*. Since the locations of events within balanced spatial clusters are plausibly assumed to be exogenous (unobserved confounders are arguably more similar within selected spatial clusters), we can further reduce bias. The choice of buffer size for Fisher’s exact test involves a trade-off: Although with narrower buffer bandwidth, the homogeneity of the distribution of the location determinant is tested more strictly, the number of observations within buffers becomes smaller, thus weakening statistical power.

### Stage 3: Analysis

The analysis stage examines the impacts of the treatment variables on the outcomes of interest based on the samples constructed in the second design stage. We estimate a regression equation controlling for spatial cluster fixed effects by ordinary least squares (OLS). We assume that within spatial clusters, there is no residual spatial variability and neighborhood spillover effects, if any, are constant. Then, in our regression model, spatial cluster fixed effects control for local spillover effects within spatial clusters. We assume that there are no spillover effects across spatial clusters. We check the sensitivity of estimation results to potential omitted variable bias due to remaining unobserved confounders within balanced spatial clusters. We do so by employing coefficient stability approaches (e.g., [18, 20]) corresponding to our regression-based framework.

#### Internal and external validity

Of critical importance in empirical studies is the validation of empirical findings [21]. Internal validity is the degree to which the findings for the (sub)population being studied are credible; external validity is the degree to which the findings can be extrapolated to other populations and settings [22]. Our framework aims to improve internal validity: The estimates based on the local sample within balanced spatial clusters have higher internal validity than those based on the global sample. On the other hand, the former estimates are prone to limited external validity because the local sample may not represent the population of interest. In quasi-experimental designs, such a trade-off always exists. Although the subpopulation being studied can differ distinctly from the population of interest, in our approach, well-defined subpopulations represented by the local sample are identified and thus assessing the credibility of findings for the population of interest is possible. This is not possible in standard instrumental variables methods because subpopulations affected by instruments are not identified.

### A case study: Impacts of the Cambodian genocide

Our case study examines how the Cambodian genocide under the Pol Pot regime (1975-1979) altered people’s post-conflict behavior, parental investment in child education in particular. The locations of events and subjects of our interest are those of execution sites (“killing sites”) and households, respectively. The relationship between violence and behavior is an important topic in psychology and social sciences [23–25]. The availability of the two types of georeferenced point data enables our framework; in contrast, finding valid instrumental variables is difficult in this context. After providing brief historical background, the motivation, and the data, we implement our GIS-based empirical research design.

#### Historical background

The Khmer Rouge (officially the Communist Party of Kampuchea) led by Pol Pot ruled Cambodia in a form of primitive communism from 1975 to 1979. Its communist revolution completely denied any right to private property, not only material private properties, but also one’s own family: Spouses and children were treated as collective property owned by the state [26]. People were forced to conform with the ideologies of the Pol Pot regime; those who disobeyed Khmer Rouge’s rules were treated as enemies of the society, being sent to reeducation camps and/or executed. During the Pol Pot era, approximately two million people died of execution, disease, starvation, or exhaustion [27].

#### Motivation

Our case study is motivated by the following social contexts. Under the Pol Pot regime, intellectuals were persecuted and many of them were executed [28]; formal school education was also denied and abolished. After its collapse in 1979, the remnants of the Khmer Rouge continued guerilla warfare against the new government army until the 1990s; thus, the threat of violence against people by the Khmer Rouge persisted. Indeed, survivors often suffered from long-term mental health disorders, such as post-traumatic stress disorder (PTSD) [29]. Although formal school education resumed soon after the regime’s collapse [30], parental investment in child education may have been influenced negatively by the Khmer Rouge’s rules, particularly for those who were strongly exposed to the genocidal violence.

#### Data

We utilize two data sets: the Khmer Rouge historical database and the complete count 1998 Cambodian Population Census microdata. The former data contain comprehensive information on the genocide during the Pol Pot era (events) with geocoded locations of more than 500 killing sites (e.g., burials, prisons) and the number of victims (marks). The latter data contain the basic information of individual and household socioeconomic characteristics (subjects’ outcomes) and geocoded locations of villages in the country. Since no location information is available at the household level, we substitute the point locations of villages for those of households; thus, all households residing in the same village share the same location information. Section 1 in S1 Supplementary Material provides a detailed description of the data. All data used in the paper were fully anonymized before we accessed them. The IRB approval for this study was received from the University of Tokyo (Approval number: 16-80, Date: August 10, 2016).

### Stage 1: Diagnosis

**Fig 1:**
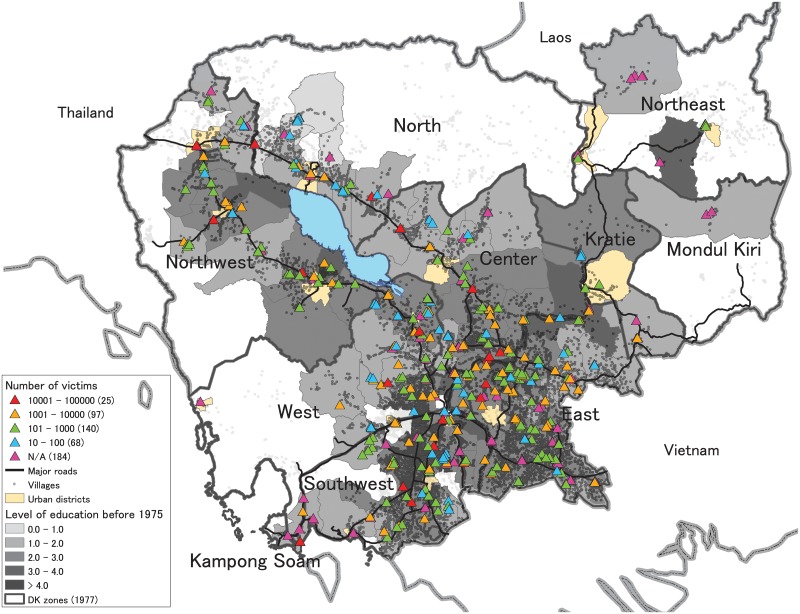
Geographic distribution of killing sites under the Pol Pot regime. The geographic distribution of 514 killing sites and their number of victims in districts surveyed by DC-Cam are depicted (districts not surveyed have a white background). The 1977 administrative zones of the Pol Pot regime (DK zones (1977)) and the 1998 districts are depicted. For information on education and major roads, see the text and Section 2 in S1 Supplementary Material.

We first examine how killing sites were established. According to anecdotal evidence provided by international organizations and historians, the Khmer Rouge used schools, universities, and government buildings as prisons and reeducation camps and trucks to transport prisoners to killing sites [31]. This suggests that the locations of killing sites were relatively developed areas. Using GIS, we plot the locations of 514 killing sites, along with information about victims, as well as the locations of villages on the base map of the 1977 administrative divisions (Fig 1). We also overlay the major roads (national and provincial road networks) in 1973 and the district mean education level of non-migrant women aged 36-50 (a cohort that should have finished primary school education, if receiving any education, before 1975; see Section 2 in S1 Supplementary Material for a detailed discussion) to capture the level of regional development before the Pol Pot era. This GIS map reveals that killing sites were commonly located near major roads and in districts with relatively high education levels (though in the eastern and western zones many sites were also located relatively far from major roads). These relationships are also confirmed empirically (Table B in S1 Supplementary Material). These results suggest that the level of regional development prior to the Pol Pot era is a key determinant of the placement of killing sites.

### Stage 2: Design

We first describe the population of interest and the sample, then create treatment variables, and after that follow the three steps of constructing a credible sample.

#### Population

In 1975, urban people, who were persecuted under the Pol Pot regime, were forced to migrate to the countryside to engage in forced agricultural work [28]. During the Pol Pot era, many of them experienced forced migration several times. Because our treatment variables (defined below) are based on the locations of killing sites and villages where couples lived in 1998, we can construct treatment variables only for non-migrant couples. The population of our interest is thus non-migrant rural couples (who had their first child during and after the Pol Pot era, as detailed below). We exclude urban couples and rural couples with migration experience (about 57% of the couples in rural areas) from the scope of our study.

#### Sample

Our global sample consists of non-migrant rural couples in the areas surveyed about the mass killings who had their first child in 1977-1982 (see Section 3.1 in S1 Supplementary Material for details). Because the couples who had their first child during and after the Pol Pot regime had distinct institutional experiences (the former were controlled as family organizations and the latter were not), we divide the sample into two groups: couples whose first child was born in 1977-1979 and couples whose first child was born in 1980-1982. Taking into account a transition period, we further divide the latter into two groups: couples whose first child was born in 1980 and those whose first child was born in 1981-1982. We examine the genocide impacts on children’s educational outcomes (defined below) for these three subsamples separately. We assume that the timing of having the first child is unrelated to potential genocide impacts (Section 3.2 in S1 Supplementary Material provides evidence for this assumption). This assumption implies that for example, if couples whose first child was born in 1980 or 1981-1982 had had their first child in 1977-1979, their estimated genocide impacts would be identical to those for couples whose first child was actually born in 1977-1979. For illustrative purposes and the sake of brevity, we highlight the results for the subsample of couples whose first child was born in 1977-1979 in the text and report those for other two subsamples in S1 Supplementary Material.

#### Treatment variables

Using GIS, we create two treatment variables of the genocidal violence. A binary measure (*Genocidal Violence I*) takes the value of 1 if the points of villages where couples resided are within 3.0 km of at least one killing site and 0 otherwise. A continuous measure (*Genocidal Violence II*) is the logarithmic value of the inverse-distance weighted sum of the number of victims for all killing sites located within 6.0 km of villages where couples resided (this corresponds to 6.0 km spatial clusters defined below) (we use log because the original value has right-skewed distribution, S1 Fig). Since 184 killing sites lack information about the number of victims (Fig 1), the continuous measure is defined only among villages with complete victim information for all killing sites located within 6.0 km of the villages. For this reason, we focus on the binary treatment variable in the design stage for the analysis of both the binary and continuous treatment variables. Section 4 in S1 Supplementary Material conducts robustness checks for alternative cluster size and continuous measures.

#### Step 1—Global sample

In the three subsamples of the global sample (*Global Sample*), we examine the joint significance of pre-treatment village characteristics (distance to major roads (km), the proportion of non-migrant women aged 36-50 with grade 1-5 of primary education and the proportion of non-migrant women aged 36-50 with grade 6 or above) and parental characteristics (mother’s and father’s age, and a set of dummy variables for mother’s and father’s educational attainment (grade 1-5 and grade 6 or above)). The dependent variable is the binary treatment variable defined above (i.e., *Genocidal Violence I*). We estimate all regression equations by OLS with zone and district fixed effects controlled for. Logistic regression with many dummy variables can have a well-known incidental parameter problem [32]. The results reported in column 1 of Table 1 (and columns 1 and 2 of Table J in S1 Supplementary Material) show that villages (couples) located (living) near killing sites are more likely to have been developed (educated) in the subsample of couples whose first child was born in 1977-1979 (the results for other two subsamples reported in columns 1 and 2 of Table J in S1 Supplementary Material are similar). Thus, controlling for these pre-treatment factors is indispensable. With limited relevant pre-treatment variables in our data, however, unobserved confounders are a major concern. It is likely that our estimates based on Global Sample suffer from omitted variable bias.

**Table 1:** Exogeneity of locations of killing sites/binary genocide measure.

Sample: Subsample:	Global Sample 1977-79	Local Sample I 1977-79	Local Sample II 1977-79
Variable	[1]	[2]	[3]
	A. Village characteristics		
Distance to major roads (km)	−0.004*** (0.001)	0.001 (0.007)	−0.010 (0.012)
Prop. of non-migrant women aged 36-50 with grade 1-5	0.118*** (0.034)	0.041 (0.073)	0.053 (0.117)
Prop. of non-migrant women aged 36-50 with grade 6 or above	0.485*** (0.064)	0.514*** (0.115)	0.054 (0.184)
Zone and district fixed effects	√	√	√
Spatial cluster fixed effects		√	√
N	3,989	1,979	839
R-squared	0.145	0.381	0.478
*p*-value of the listed variables	0.000	0.000	0.755
	B. Parental characteristics		
Mother’s age	0.000 (0.002)	−0.002 (0.002)	0.000 (0.004)
Father’s age	0.000 (0.001)	−0.003* (0.001)	−0.003 (0.002)
Mother with grade 1-5	0.042*** (0.010)	0.025* (0.014)	0.034 (0.022)
Mother with grade 6 or above	0.065*** (0.017)	0.058*** (0.022)	0.047 (0.035)
Father with grade 1-5	0.023** (0.011)	0.000 (0.016)	−0.012 (0.027)
Father with grade 6 or above	0.058*** (0.013)	0.018 (0.018)	−0.010 (0.028)
Zone and district fixed effects	√	√	√
Spatial cluster fixed effects		√	√
N	11,141	5,738	2,137
R-squared	0.149	0.407	0.477
*p*-value of the listed variables	0.000	0.003	0.538
Num. of killing sites	435	433	115

The table reports OLS estimates where the unit of observation is the village in panel A and the household in panel B. Robust standard errors are reported in parentheses in panel A and robust standard errors, adjusted for clustering by village, are reported in parentheses in panel B. See the text for description of the samples. The dependent variable is an indicator variable equal to 1 if villages are located within 3.0 km of killing sites and 0 otherwise. *p*-values are from *F*-tests for the joint significance of the listed variables.

*** *p* < 0.01;

** *p* < 0.05; and

* *p* < 0.1.

#### Step 2—Spatial clusters

**Fig 2:**
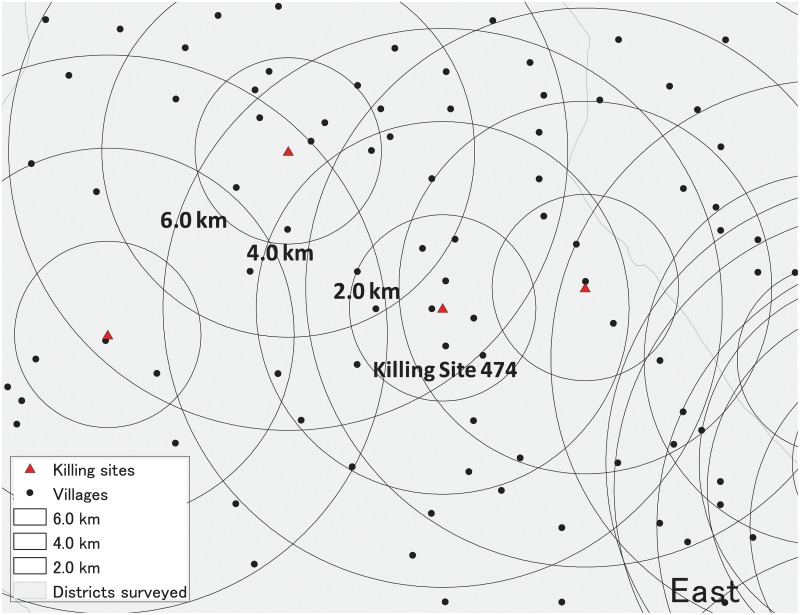
Spatial clusters. The number of villages within 0-2.0 km, 2.0-4.0 km, and 4.0-6.0 km buffers of Killing Site 474 is 9, 15, and 23, respectively.

Using GIS, we create 6.0 km spatial clusters around the locations of killing sites and identify village points within the spatial clusters (Fig 2). We then limit the sample to couples living within the spatial clusters. We assess this local sample (*Local Sample I*) in the same way as we do Global Sample above, except that *spatial cluster fixed effects* are additionally controlled (433 spatial clusters in total): Unobserved confounders common within spatial clusters are now fully controlled for (see S2 Fig for the distribution of the number of spatial clusters to which villages in the three subsamples of all local samples belong). The results reported in column 2 of Table 1 (and columns 3 and 4 of Table J in S1 Supplementary Material) show that although some significant differences disappear, substantial significant differences remain; the listed variables are all jointly significant in the three subsamples (in both panels A and B). These results raise a concern that our estimates in Local Sample I are likely to still suffer from omitted variable bias because unobserved confounders that are related to the observed covariates may vary within the spatial clusters.

#### Step 3—Balanced spatial clusters

We further limit the sample to couples living within selected spatial clusters with similar levels of regional development according to Fisher’s exact test. Since it is not feasible to implement Fisher’s exact test for the two measures depicted in Fig 1, we use the proportion of migrant households as a proxy for the level of regional development (see Section 3.3 in S1 Supplementary Material for details); in-migration is generally strongly correlated with regional development [33]. With the lack of historical migration data, we use data from the 1998 Census to calculate the migrant proportion. We confirm that the migrant proportion is positively correlated with the locations of killing sites (Table B in S1 Supplementary Material). We assume that this measure captures the level of regional development within spatial clusters well.

Within each 6.0 km spatial cluster, we create smaller buffers using a 2.0 km bandwidth which is narrower than the 3.0 km bandwidth determining the treatment status (Fig 2). For each of the three subsamples of Local Sample I, we conduct Fisher’s exact test on the homogeneity in the proportion of migrant households across the three (0-2.0 km, 2.0-4.0 km, and 4.0-6.0 km) buffers. We define spatial clusters as balanced if the null hypothesis of no association in the proportion of migrant households across the three buffers cannot be rejected for all three subsamples. To be conservative, we also conduct the same exercise for 4.0 km spatial clusters.

To provide a specific example, Table 2 reports the results of Fisher’s exact tests for Killing Site 474 depicted in Fig 2 (see S3 Fig for the location of Killing Site 474). In all three subsamples, the number of non-migrant households is larger than that of migrant households within the three buffers. As none of the three subsamples can reject the null hypothesis at conventional levels, the spatial cluster of Killing Site 474 is balanced (the same results hold for the 4.0 km spatial cluster (not reported)). S3 Fig shows the results of the same Fisher’s exact tests for all 514 killing sites. There are 115 balanced spatial clusters (see Section 3.3 in S1 Supplementary Material for details). Although the sample is limited to households within 115 balanced spatial clusters, we also adjust for spatial cluster fixed effects for unbalanced spatial clusters to which they belong, if any, in our regression model (see panel B of S2 Fig).

**Table 2:** Results of Fisher’s exact tests for killing site 474.

	No. of Villages	Non-migrant HHs	Migrant HHs	Total
	[1]	[2]	[3]	[4]
	A. 1977-79 (*p*-value = 0.578)			
0-2.0 km	6	9	2	11
2.0-4.0 km	9	19	11	30
4.0-6.0 km	16	22	9	31
Total	31	50	22	72
	B. 1980 (*p*-value = 0.638)			
0-2.0 km	9	11	6	17
2.0-4.0 km	10	15	5	20
4.0-6.0 km	14	18	12	30
Total	33	44	23	67
	C. 1981-82 (*p*-value = 0.704)			
0-2.0 km	8	17	6	23
2.0-4.0 km	14	28	11	39
4.0-6.0 km	20	37	20	57
Total	42	82	37	119

*p*-values are from two-sided Fisher’s exact tests.

Almost all significant differences found in Global Sample and Local Sample I vanish in this further selected sample (*Local Sample II*) based on these 115 balanced spatial clusters (Table 1 column 3 and Table J columns 5 and 6). The magnitude of many coefficients becomes smaller. In addition, the listed variables are jointly insignificant at conventional levels in all regressions. These results suggest that the observed covariates and unobserved covariates related to the observed ones, if any, are similar within the balanced spatial clusters; thus, Local Sample II should yield less biased estimates than Global Sample and Local Sample I. At the same time, relative to the population of interest, Local Sample II (and Local Sample I) contain households and villages with favorable characteristics because they focus on villages around killing sites, which tend to have been located in relatively developed areas (see Section 3.1 in S1 Supplementary Material for a more detailed consideration). Therefore, the results should be taken with some caution regarding external validity.

### Stage 3: Analysis

We construct outcome variables, specify regression models, and present the estimation results.

#### Outcomes

We consider educational outcomes for children aged 15-21 and 6-14. In the 1998 Cambodian education system, the former cohort had already finished the nine-year compulsory education (due to delayed entry, temporary dropout, or grade retention, some were still receiving it though), and the latter cohort were still receiving it, if they were receiving any education. Most of these children were born after the Pol Pot regime. The exposure of couples to the genocidal violence may have affected their fertility decisions and thus the existence of most of these children. Thus, we use household, not child, as a unit of analysis. The household-level outcome measures of interest are the average years of schooling (*Years of schooling*) for children aged 15-21 and the average grade progression (*Grade progression*) for children aged 6-14, where the grade progression of each child is given by *Grade*−(*Age*−5), which takes 0 if the child progresses from any grade to the next higher grade and negative values otherwise. Table D in S1 Supplementary Material provides the descriptive statistics of these outcome measures.

#### Empirical specification

We estimate the following regression model,

$$\begin{array}{c} Y_{ivbdz}=\alpha +\gamma GenocidalViolence_{v}+X_{i}^{{}^{\prime }}\beta_{1}+X_{v}^{{}^{\prime }}\beta_{2}+\phi_{b}+\pi_{d}+\lambda_{z}+\epsilon_{ivbdz}, \end{array}$$ (1)

where *Y*_*ivbdz*_ is an educational outcome of household *i* in village *v*, spatial cluster *b*, district *d*, and zone *z*; *GenocidalViolence*_*v*_ is the binary/continuous genocide measure; the vectors *X*_*i*_ and *X*_*v*_ include parental and village characteristics (listed in Table 1), respectively; *ϕ*_*b*_ denotes spatial cluster fixed effects (only for local samples); *π*_*d*_ and *λ*_*z*_ denote district and zone fixed effects, respectively. Some spatial clusters are located over more than one district or zone. District and zone fixed effects, respectively, adjust for district- and zone-level unobserved confounders, if any. A parameter of our interest is *γ*, which captures the effect of the genocidal violence on the outcome. We estimate this model by OLS with robust standard errors clustered by village (at the level of which the treatment variables are measured). The estimates based on Local Sample II should be least biased.

#### Results

**Fig 3:**
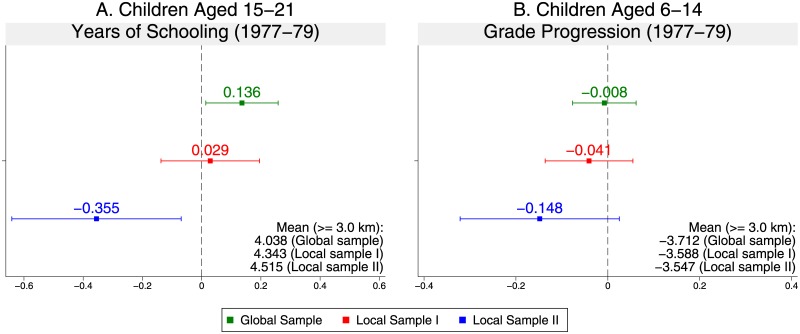
Point estimates and 95% confidence intervals of genocide impacts on children’s educational outcomes (binary genocide measure).

Fig 3 plots the point estimates and 95% confidence intervals of the impacts of the binary genocide measure for children aged 15-21 (panel A) and 6-14 (panel B) of the couples whose first child was born in 1977-1979. For comparison, we present all results in Global Sample and Local Samples I and II; the smaller the sample size, the wider the confidence intervals. Although the point estimates are mostly positive in Global Sample and Local Sample I, estimates become negative in Local Sample II. For example, in Local Sample II, although the couples’ exposure to the genocidal violence increased the years of schooling of children aged 15-21 by 0.136 years in Global Sample, it rather decreased their years of schooling by 0.355 years (7.9% of the mean among those with no exposure). Such adverse impacts are found for children aged both 15-21 and 6-14. In contrast, among couples whose first child was born in 1980 or 1981-1982, none of the estimates in Local Sample II are statistically significantly different from 0 (see S4 Fig).

**Fig 4:**
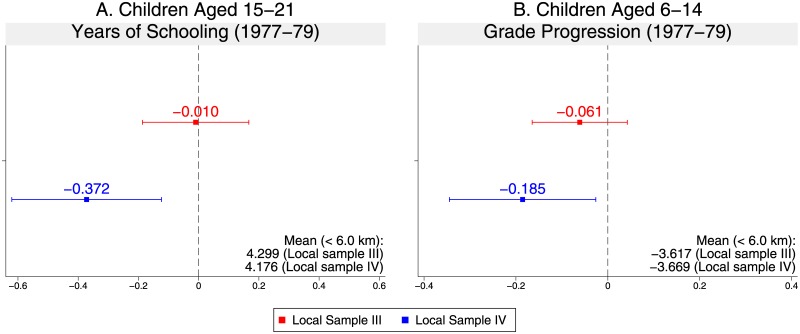
Point estimates and 95% confidence intervals of genocide impacts on children’s educational outcomes (continuous genocide measure).

Fig 4 (and S5 Fig) show the results for the continuous genocide measure in Local Sample I with victim information (*Local Sample III*) and Local Sample II with victim information (*Local Sample IV*), which contain 289 spatial clusters and 83 balanced spatial clusters, respectively (Table A in S1 Supplementary Material). To construct *Local Sample IV* from *Local Sample III*, we employ the same procedure of Fisher’s exact test discussed above. The results are qualitatively the same as those for the binary genocide measure.

Regression diagnostics based on Local Samples II and IV suggest that our models are appropriate (see S6 and S7 Figs). It is also noted that we use robust standard errors adjusted for clustering by village for conservative statistical inference.

To address remaining threats to internal validity, we assess the sensitivity of the results to potential omitted variable bias due to unobserved confounders that vary within balanced spatial clusters for Local Samples II and IV, using the Oster’s coefficient stability approach [18] (see Section 5 in S1 Supplementary Material for details). The results confirm that the estimated negative impacts are robust to omitted variable bias even under conservative assumptions.

Thus, we conclude that the genocidal violence had adverse impacts on child education among the couples who had their first child during the Pol Pot era. The analysis and discussion of potential mechanisms underlying these patterns are provided elsewhere [34].

## Concluding remarks

This paper provided a regression-based framework for GIS-based empirical research design using georeferenced point data for both spatial events of interest and subjects exposed to the events and illustrated its utility and implementation through an empirical case study from Cambodia. GIS is particularly useful in understanding the locational determinants of spatial events, creating treatment variables, constructing credible samples, and implementing blocking (local control). GIS can potentially play a key role in designing credible and transparent empirical research as spatial point data become more widely available. Much work is needed to overcome the limitations of our approach. Promising avenues include designing the optimal choice of the size of spatial clusters and the bandwidth of buffers, allowing a change in subject locations, capturing local spillover effects, and analyzing dynamic treatment effects. Ultimately, our approach needs to be extended to one in the potential outcomes framework for causal analysis. These works are left for future research.

## Supporting information

S1 Fig Distribution of continuous genocide measures. Kernel density of the distribution of the continuous genocide measures based on the first-, second-, and third-order polynomials in distance is shown for each subsample in Global Sample.
(PDF)

S2 Fig Distribution of number of spatial clusters to which villages belong. The figure provides the distribution of the number of spatial clusters to which villages in each subsample of Local Samples I (panel A), II (panel B), III (panel C), and IV (panel D) belong.
(PDF)

S3 Fig Results of Fisher’s exact tests. The figure provides the results of Fisher’s exact tests. The location of killing site 474 analyzed in Fig 2 and Table 2 is depicted.
(PDF)

S4 Fig Estimation results (binary genocide measure)—Other subsamples. The figure provides point estimates and 95% confidence intervals of genocide impacts on children’s educational outcomes based on binary genocide measure.
(PDF)

S5 Fig Estimation results (continuous genocide measure)—Other subsamples. The figure provides point estimates and 95% confidence intervals of genocide impacts on children’s educational outcomes based on continuous genocide measure.
(PDF)

S6 Fig Regression diagnostics—Local Sample II (binary genocide measure). Regression diagnostics are presented for each subsample of Local Sample II. Each of the three figures depicts the following: the distribution of residuals, along with a normal density (green) (left); a normal quantile-quantile plot of residuals (middle); a residual plot, along with a locally weighted scatterplot smoothing curve (bandwidth = 0.8) (green) (right).
(PDF)

S7 Fig Regression diagnostics—Local Sample IV (continuous genocide measure). Regression diagnostics are presented for each subsample of Local Sample IV. See the notes to S6 Fig for each figure.
(PDF)

S1 Supplementary Material PDF file containing the Supplementary Material. This file provides the detailed descriptions of data, diagnosis, design, and analysis (Section 1: Data details; 2: Diagnosis details; 3: Design details; 4: Robustness checks; 5: Sensitivity analysis), including 18 tables (Tables A–R).
(PDF)

S1 File Replication files. This zip file contains the dataset and Stata do-file to replicate the empirical analyses presented in the case study.
(ZIP)
