# Supplementary figures and images for: GIS for empirical research design: An illustration with georeferenced point data
Source: PLoS One. 2019 Mar 4;14(3):e0212316. doi: 10.1371/journal.pone.0212316 (PMC6398843; doi:10.1371/journal.pone.0212316)

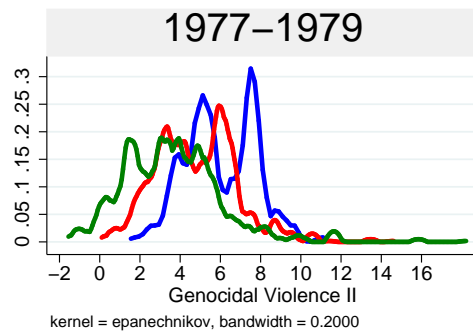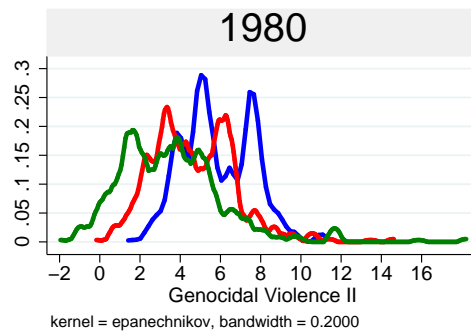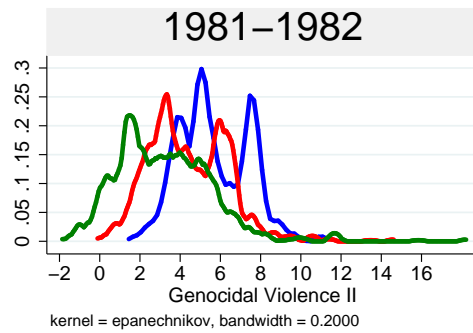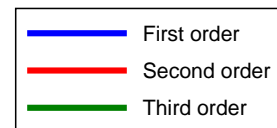

Supplement: S1 Fig — Kernel density of the distribution of the continuous genocide measures based on the first-, second-, and third-order polynomials in distance is shown for each subsample in Global Sample. (PDF) [file pone.0212316.s001.pdf]

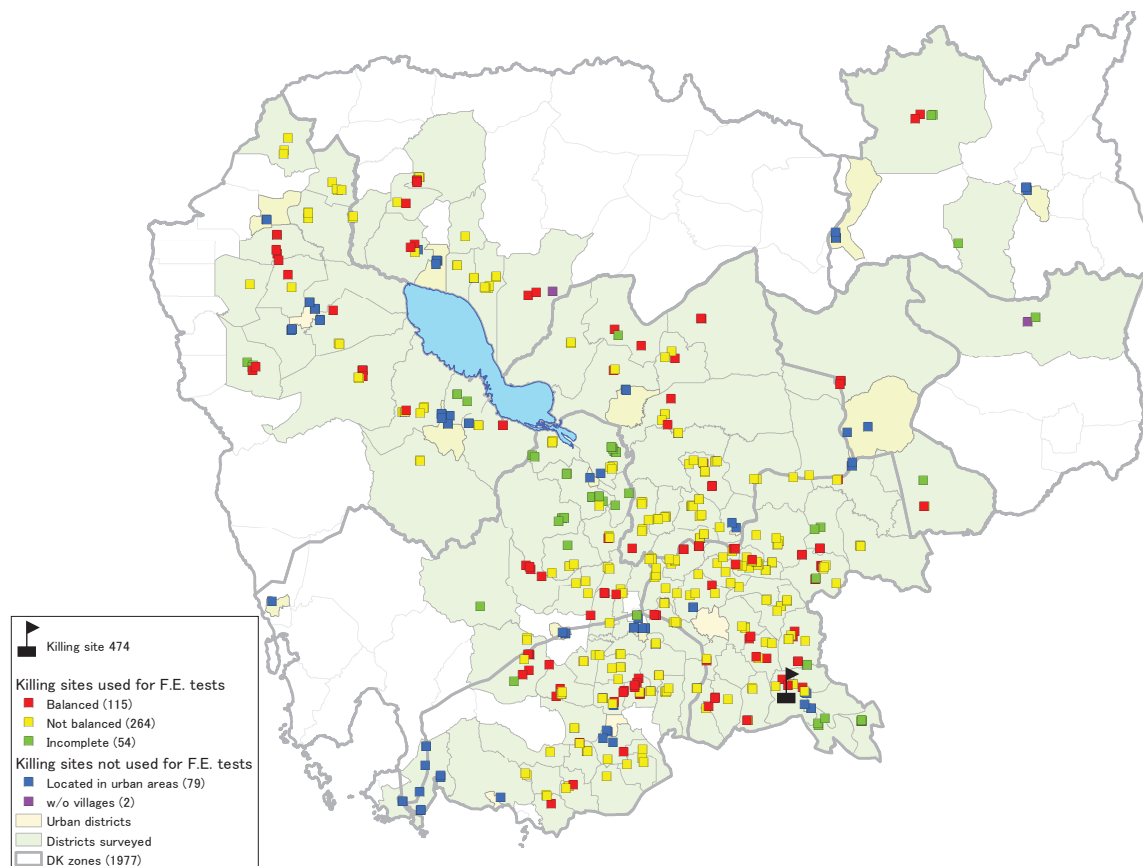

Supplement: S3 Fig — The figure provides the results of Fisher’s exact tests. The location of killing site 474 analyzed in Fig 2 and Table 2 is depicted. (PDF) [file pone.0212316.s003.pdf]

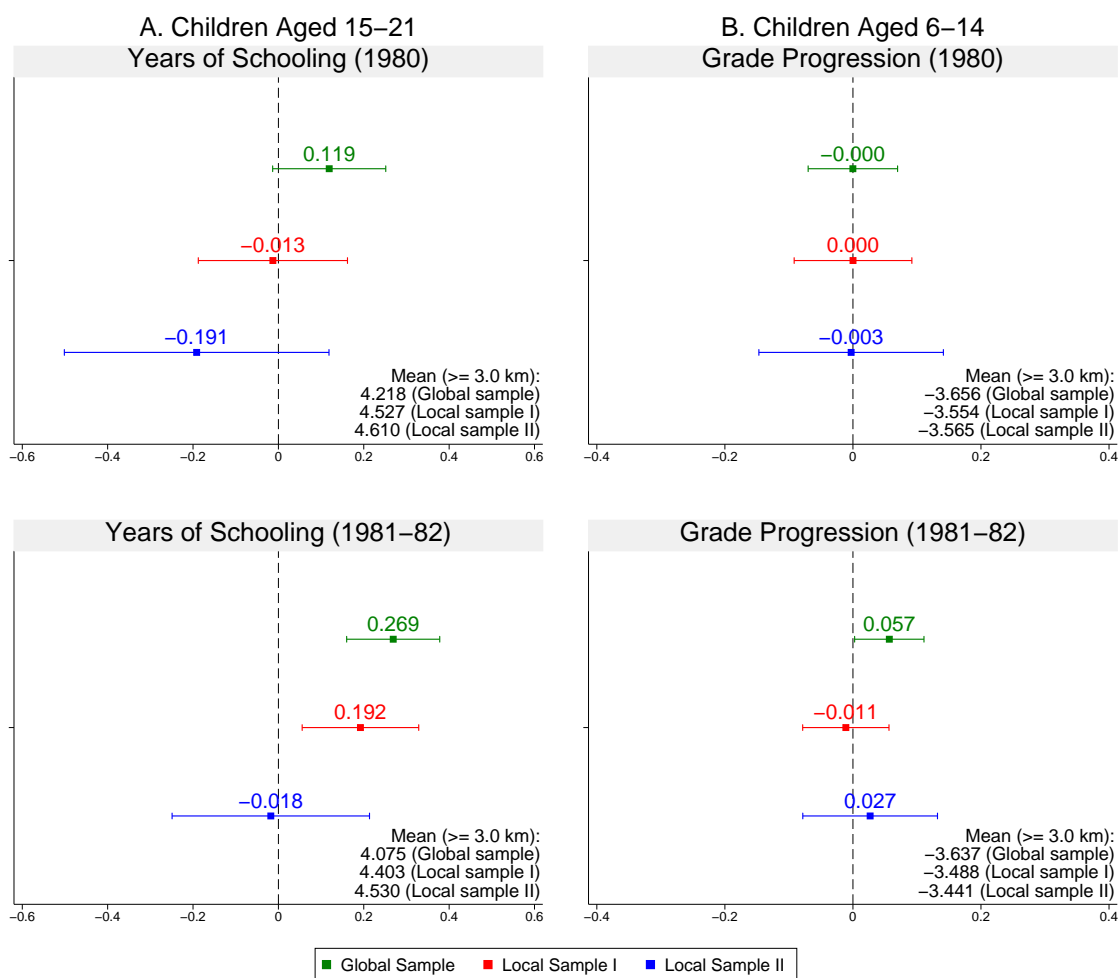

Supplement: S4 Fig — The figure provides point estimates and 95% confidence intervals of genocide impacts on children’s educational outcomes based on binary genocide measure. (PDF) [file pone.0212316.s004.pdf]

## A. Children Aged 15–21; Years of Schooling

1977–79

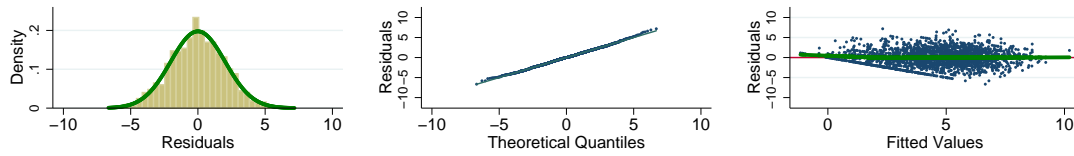

1980

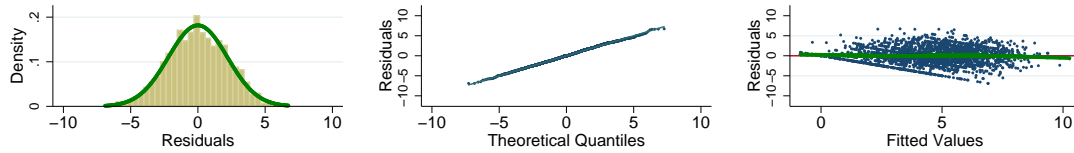

1981–82

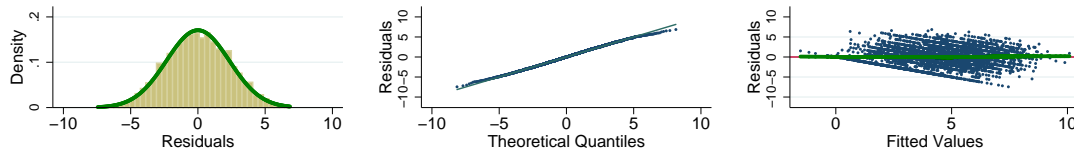

## B. Children Aged 6–14; Grade Progression

1977–79

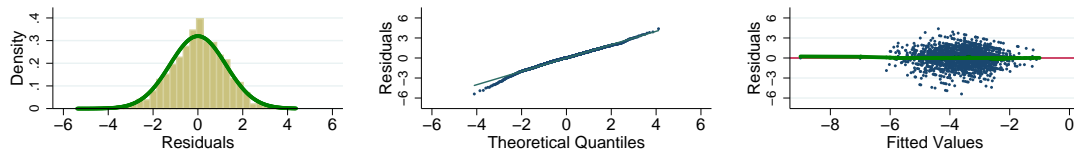

1980

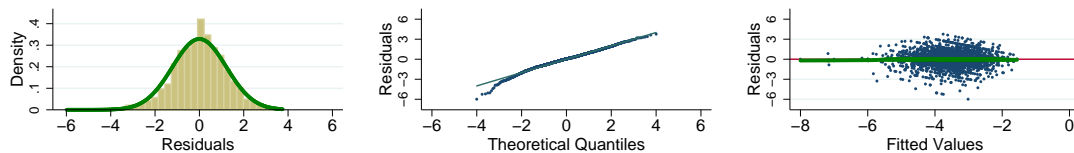

1981–82

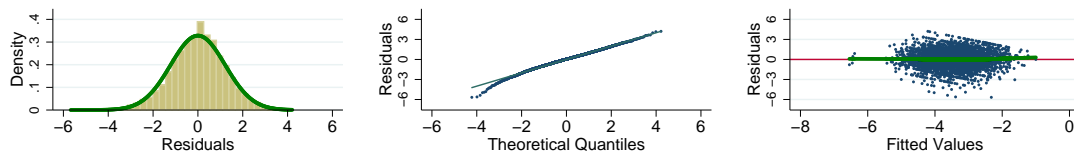

Supplement: S6 Fig — Regression diagnostics are presented for each subsample of Local Sample II. Each of the three figures depicts the following: the distribution of residuals, along with a normal density (green) (left); a normal quantile-quantile plot of residuals (middle); a residual plot, along with a locally weighted scatterplot smoothing curve (bandwidth = 0.8) (green) (right). (PDF) [file pone.0212316.s006.pdf]

## A. Children Aged 15–21; Years of Schooling

1977–79

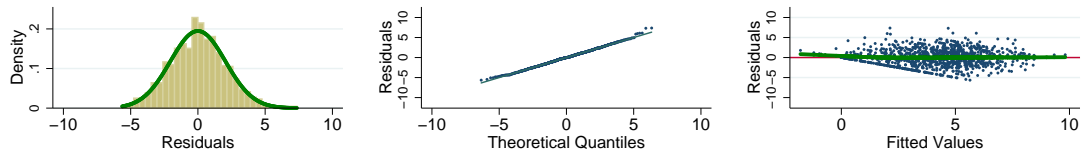

1980

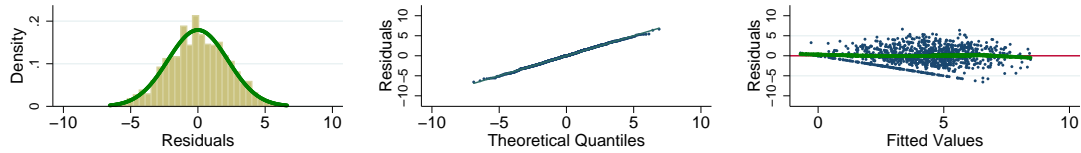

1981–82

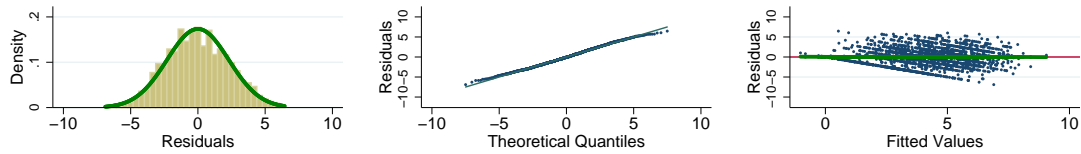

## B. Children Aged 6–14; Grade Progression

1977–79

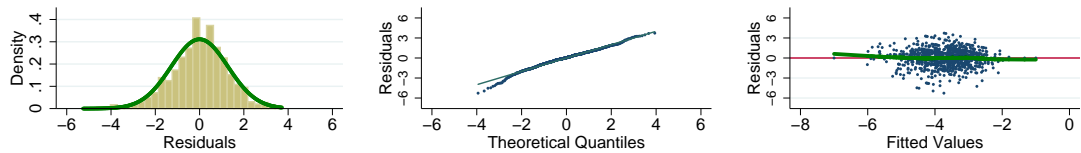

1980

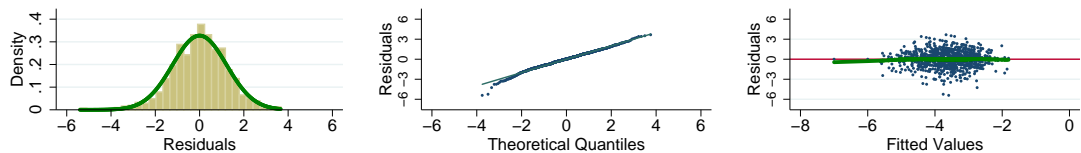

1981–82

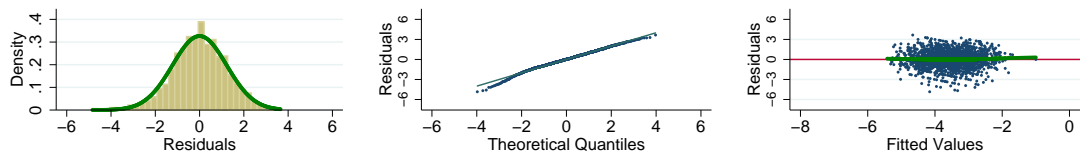

Supplement: S7 Fig — Regression diagnostics are presented for each subsample of Local Sample IV. See the notes to S6 Fig for each figure. (PDF) [file pone.0212316.s007.pdf]
