# Supplementary material for: GIS for empirical research design: An illustration with georeferenced point data
Source: PLoS One. 2019 Mar 4;14(3):e0212316. doi: 10.1371/journal.pone.0212316 (PMC6398843; doi:10.1371/journal.pone.0212316)

A. Local Sample I (all spatial clusters)

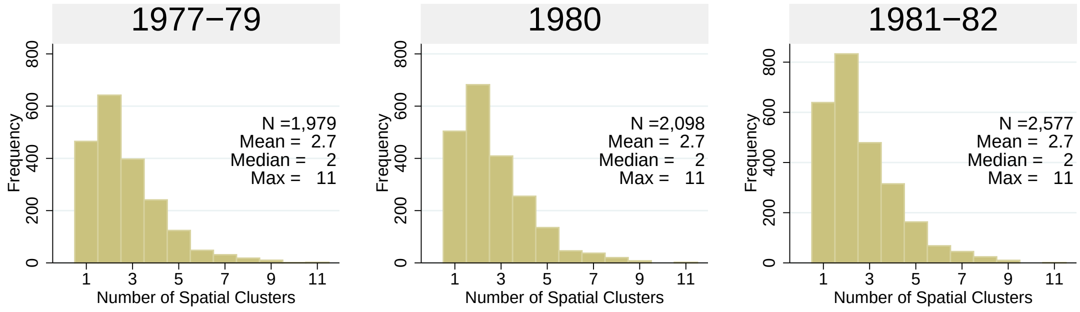

B. Local Sample II (balanced spatial clusters)

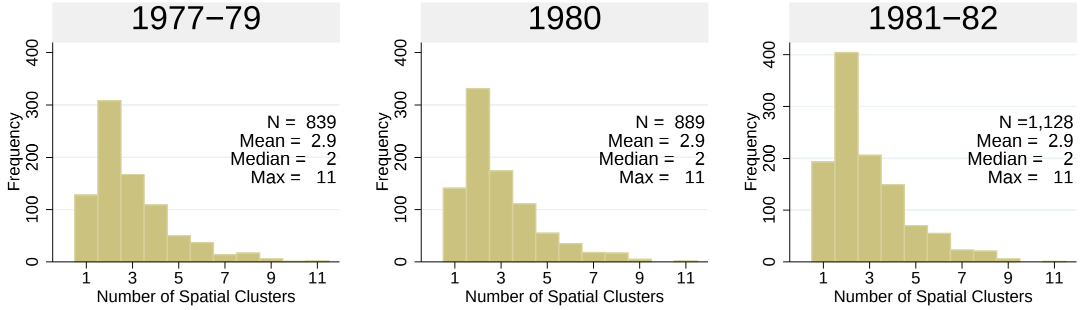

C. Local Sample III (all spatial clusters)

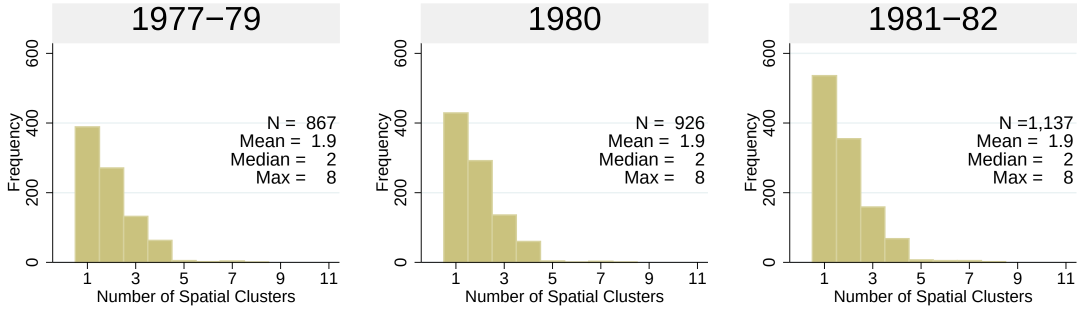

D. Local Sample IV (balanced spatial clusters)

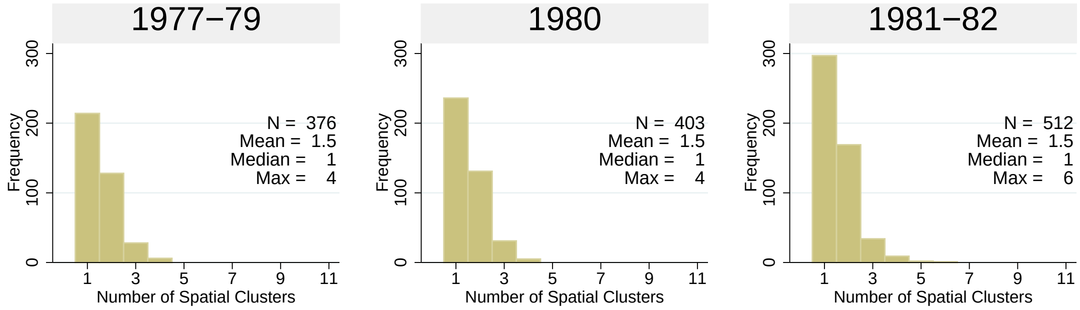

Supplement: S2 Fig — The figure provides the distribution of the number of spatial clusters to which villages in each subsample of Local Samples I (panel A), II (panel B), III (panel C), and IV (panel D) belong. (PDF) [file pone.0212316.s002.pdf]
