# Supplementary material for: GIS for empirical research design: An illustration with georeferenced point data
Source: PLoS One. 2019 Mar 4;14(3):e0212316. doi: 10.1371/journal.pone.0212316 (PMC6398843; doi:10.1371/journal.pone.0212316)
Supplement: S5 Fig — The figure provides point estimates and 95% confidence intervals of genocide impacts on children’s educational outcomes based on continuous genocide measure. (PDF) [file pone.0212316.s005.pdf]

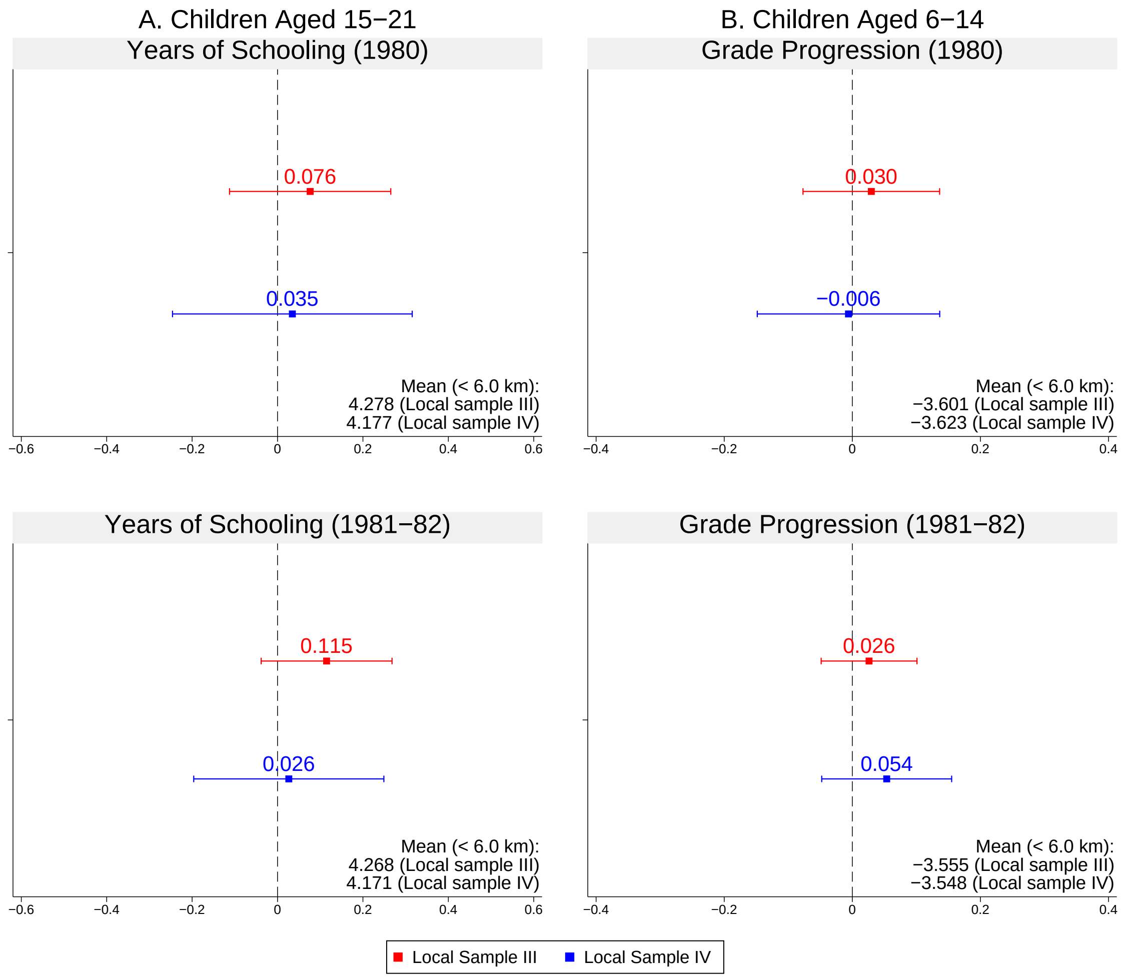
A. Children Aged 15−21
Years of Schooling (1980)
0.076
0.035
Mean (< 6.0 km):
4.278 (Local sample III)
4.177 (Local sample IV)
−0.6
−0.4
−0.2
0
0.2
0.4
0.6
B. Children Aged 6−14
Grade Progression (1980)
0.030
−0.006
Mean (< 6.0 km):
−3.601 (Local sample III)
−3.623 (Local sample IV)
−0.4
−0.2
0
0.2
0.4
Years of Schooling (1981−82)
0.115
0.026
Mean (< 6.0 km):
4.268 (Local sample III)
4.171 (Local sample IV)
Grade Progression (1981−82)
0.026
0.054
Mean (< 6.0 km):
−3.555 (Local sample III)
−3.548 (Local sample IV)
Local Sample III
Local Sample IV
