# Supplementary material for: GIS for empirical research design: An illustration with georeferenced point data
Source: PLoS One. 2019 Mar 4;14(3):e0212316. doi: 10.1371/journal.pone.0212316 (PMC6398843; doi:10.1371/journal.pone.0212316)
Supplement: S1 Supplementary Material — This file provides the detailed descriptions of data, diagnosis, design, and analysis (Section 1: Data details; 2: Diagnosis details; 3: Design details; 4: Robustness checks; 5: Sensitivity analysis), including 18 tables (Tables A–R). (PDF) [file pone.0212316.s008.pdf]

Supplementary Material

GIS for Empirical Research Design: An Illustration with Georeferenced Point Data

Katsuo Kogure* Yoshito Takasaki†
Kyoto University University of Tokyo

February 22, 2019

Contents

1 Data details **A–3**

1.1 Genocide data . . . A–3

1.2 Census data . . . A–3

2 Diagnosis details **A–3**

3 Design details **A–4**

3.1 Study population, samples, and external validity . . . A–4

3.2 Exogeneity of the timing of childbearing . . . A–5

3.3 Fisher's exact test . . . A–5

4 Robustness checks **A–6**

5 Sensitivity analysis **A–7**

List of Tables

A Means and standard deviations – killing-site characteristics . . . A–10

B Location determinants of killing sites . . . A–11

C Construction of global sample . . . A–12

D Means and standard deviations – household characteristics . . . A–13

E Means and standard deviations – village characteristics . . . A–16

F Treatment-control mean differences – household characteristics . . . A–17

G Treatment-control mean differences – village characteristics . . . A–20

H External validity – mean differences across samples . . . A–21

*Email: katsukogure@gmail.com.
†Email: takasaki@e.u-tokyo.ac.jp.

I Exogeneity of the timing of childbearing – difference in number of children across subsamples A–24
J Exogeneity of locations of killing sites/binary genocide measure – other subsamples A–25
K Construction of sample for Fisher's exact tests A–26
L Killing-site characteristics with balanced spatial clusters and information about victims A–27
M Impacts of genocide on children's educational outcomes A–28
N Robustness check – alternative size of spatial clusters (4.0 km) A–29
O Robustness check – alternative size of spatial clusters (8.0 km) A–30
P Robustness check – alternative continuous genocide measures A–31
Q Sensitivity analysis – Local Sample II (binary genocide measure) A–32
R Sensitivity analysis – Local Sample IV (continuous genocide measure) A–33

1 Data details

1.1 Genocide data

The genocide data come from the Khmer Rouge historical database, which was developed by the Document Center of Cambodia (DC-Cam), an independent Cambodian research institute founded by Yale University's Cambodian Genocide Program in January 1995. To comprehend the mass killings during the Pol Pot era, DC-Cam conducted a large-scale survey between 1995 and 2004 covering 121 districts in 21 provinces out of 183 districts in 24 provinces in the country. The survey found 534 execution sites (killing sites) in total and collected information of their locations (latitude and longitude coordinates), types, and number of remains of victims of execution. Location information is missing for 1 site and inconsistent for 19 sites. Excluding these 20 sites, we consider the remaining 514 killing sites in our analysis.[1] Table A presents the descriptive statistics of killing sites.

1.2 Census data

The 1998 Population Census microdata, which were developed by the National Institute of Statistics, Ministry of Planning, Cambodia, contain the basic information of individual and household socioeconomic characteristics and the information of locations (latitude and longitude) of villages in the country. The data cover 12,702 village points in all provinces but Phnom Penh, the nation's capital. We substitute the 1998 village point data for those during the Pol Pot era, because the distribution of villages in 1998 is largely consistent with that during the Pol Pot era: When we select all village codes of individuals born before 1974 (one year before the Pol Pot regime began), whose birth villages are the same as those where they lived in 1998, 81% of these villages are matched with the 1998 villages.

2 Diagnosis details

Table B considers the location determinants of killing sites. The level of regional development prior to the Pol Pot era is proxied by the education levels of non-migrant women, but not men, aged 36-50. Compared to women, men were relatively more affected by armed conflict

[1]These 514 killing sites consist of seven types: 271 'burial,' 104 'prison,' 63 'burial-and-prison,' 48 'memorial,' 14 'burial-and-memorial,' 3 'memorial-and-prison,' and 11 'burial-memorial-and-prison sites'. Executions were conducted in all these types.

during the Vietnam War and were more likely to migrate or join the Khmer Rouge. Women who speak Khmer as their first language and are Buddhist are considered.

3 Design details

3.1 Study population, samples, and external validity

Study population. To define the study population using the 100% count 1998 Population Census microdata, we follow the following procedure (see Table C for the complete procedure). First, we select households with a mother aged 34-45 whose first child was born in 1977-1982; most of them were married during or right after the Pol Pot era [1]. Second, among these households, we select households that resided in rural areas in 1998 and included a husband and wife who had never migrated, i.e., non-migrant rural households. Third, we further restrict these non-migrant rural households so that unobserved factors that may be correlated with children's educational outcomes are reduced. Lastly, we exclude households with incomplete information for the variables used in our analysis. In total, we impost 17 conditions. As a result, the study population consists of 43,535 households:

(0) Study population (SP) – 43,535 households residing in districts surveyed and not surveyed by DC-Cam.

Samples. From this study population, we construct four samples as follows:

(1) Global sample (GS) – 41,054 households residing in districts surveyed by DC-Cam;

(2) Local sample I (LS I) – 20,956 households residing in villages within 6.0 km of killing sites;

(3) Local sample II (LS II) – 8,302 households residing in villages within 6.0 km of selected killing sites (6.0 km balanced spatial clusters);

(4) Local sample III (LS III) – 9,105 households residing in villages within 6.0 km of killing sites with complete victim information;

(5) Local sample IV (LS IV) – 3,821 households residing in villages within 6.0 km of selected killing sites (6.0 km balanced spatial clusters) with complete victim information.

We divide each sample into three subsamples: households whose first child was born in 1977-1979, 1980, and 1981-1982. Tables D and E present the descriptive statistics of

household and village characteristics, respectively. Table F (G) examines the difference in mean characteristics between households (villages) within 3.0 km of killing sites and those not within 3.0 km of killing sites (this corresponds to the binary treatment variable defined in the text).

External validity. Table H compares household and village characteristics across samples. Compared to SP, GS, LS I, and LS II, especially the latter two, contain households and villages with better characteristics. This is because LS I, and LS II focus on villages around killing sites, which tended to be located in relatively developed areas during the Por Pot era (Table B). Village characteristics of LS IV are slightly worse than those of LS II. This is probably because relatively developed villages are located in more than one spatial cluster, thus being likely to involve missing victim data. Household and village characteristics of LS IV are similar to those of SP.

3.2 Exogeneity of the timing of childbearing

To assess the plausibility of the assumption of exogeneity regarding the timing of childbearing, we compare the number of children among the three subsamples of LSs II and IV. The results reported in Table I show that the earlier couples had their first child, the more children they had and the number of children decreases linearly across the three subsamples. This provides evidence that fertility behaviors were similar among these couples.[2] Consistently, the three subsamples of LSs II and IV have similar covariate distributions (the results are available from the authors upon request).

3.3 Fisher's exact test

Proxy for the level of regional development. Table K provides the complete procedure for constructing the sample for Fisher's exact test. The resulting sample consists of 108,518 households. As a proxy for the level of regional development, we use the migrant proportion among nuclear households (a different sample from one for the analysis) to attain better balance. It is not feasible to use the education levels of non-migrant women aged 36-50 or the distance from villages to main roads during the Pol Pot era as a proxy

[2] According to the Cambodia Demographic and Health Survey 2000 [1], the median age at first birth is similar across cohorts: 21.5, 22.3, 21.6, 22.4, and 21.4 years old for women aged 25-29, 30-34, 35-39, 40-44, and 45-49, respectively. The last three cohorts mostly correspond to our sample (women aged 34-45 in 1998).

as we did in Fig 1, for the following reasons. First, to directly use the former, we need to consider the joint distribution of the education levels and ages, because they are systematically related to each other, but doing so is technically difficult in Fisher's exact test for the independence/homogeneity between two categorical variables [2]. Second, depending on the locations of villages relative to the main road within spatial clusters, the distance to the main road is strongly correlated with the distance from villages to the killing sites.

Results. The results of the Fisher's exact tests for all 514 killing sites are depicted in S3 Fig. The tests are not conducted for 79 sites located in urban areas which are not covered in our study and 2 sites for which no villages exist within the corresponding spatial clusters. Of the remaining 433 sites, the test results for 6.0 km spatial clusters show that 124 have balanced spatial clusters. The remaining 309 sites which do not have balanced spatial clusters include 54 sites for which the Fisher's exact tests are not done in a complete way because either one or two of the three subsamples have no observations. Spatial clusters located away from major roads during the Pol Pot era are more likely to be balanced (column 1 of Table L). Of these 124 sites with balanced spatial clusters, 9 do not have 4.0 km balanced spatial clusters. Excluding these 9 sites, LS II consists of households living within the remaining 115 balanced spatial clusters.

4 Robustness checks

We examine how robust our results reported in Table M are to alternative size of spatial clusters and alternative genocide measures. More robustness checks are available elsewhere [3].

Alternative size of spatial clusters. Tables N and O consider 4.0 km and 8.0 km spatial clusters, respectively. The estimation results are largely consistent with the original results based on the 6.0 km spatial clusters.

Alternative genocide measures. Table P considers alternative continuous treatment variables – second- and third-order polynomial in distance from villages to killing sites – for our main samples based on the 6.0 km spatial clusters (S1 Fig shows the distribution of these alternative measures along with the original linear measure). With a higher order polynomial in distance, the weights for distant villages decrease more rapidly. The estimation results are largely consistent with the original results.

5 Sensitivity analysis

Tables Q and R assess the sensitivity of the results for LS II and IV, respectively. We first consider the coefficient stability of the estimated genocide impacts to removing observed covariates (panel A). For comparison, we report the results based on the baseline model (*Baseline model*; *BM*), which controls for age (mother's age and father's age), education (a set of dummy variables for mother's and father's educational attainment (grade 1-5 and grade 6 or above)), village characteristics (the distance to major roads (km), the proportion of non-migrant women aged 36-50 with grade 1-5, and the proportion of non-migrant women aged 36-50 with grade 6 or above), and regional fixed effects (zone and district fixed effects). Spatial cluster fixed effects are always controlled for. From the baseline model, we sequentially exclude the covariates of village characteristics (*Restricted model I*), education (*Restricted model II*), and both village characteristics and education (*Restricted model III*), and age, village characteristics, and education (*Restricted model IV*; RM_{IV}); Restricted model IV controls for regional fixed effects only. The estimated coefficients are relatively stable across the five models, although statistical significance found for the couples who had their first child in 1977-1979 in LS II disappears as observed covariates are removed.

We next conduct an alternative sensitivity analysis (panel B) using the approach proposed by Oster (forthcoming) [4], building on the methodology of Altonji, Elder, and Taber (2005) [5]. This approach considers both coefficient movements and R-squared movements when covariates are included, with an assumption that both observed and unobserved covariates explain the same amount of variability (variance) in the outcome variable in a regression model. Based on her results, we consider the following bias-adjusted genocide impacts,

$$\gamma^* \approx \hat{\gamma}_{BM} - \delta(\hat{\gamma}_{RM_{IV}} - \hat{\gamma}_{BM})\frac{R^2_{max} - R^2_{BM}}{R^2_{BM} - R^2_{RM_{IV}}},$$

where $\hat{\gamma}_{BM}$ and $\hat{\gamma}_{RM_{IV}}$ are the estimates based on the Baseline model and Restricted model IV, respectively. Our interest is how coefficient estimates change due to unobserved confounders conditional on regional fixed effects, as well as spatial cluster fixed effects. δ is the proportional degree of selection. Following her suggestion, we assume equal selection: The ratio of the coefficient movement is the same as that of the R-squared movement. To allow the true genocide impacts to be overestimated or underestimated, we consider two cases for

$\delta = 1$ and $\delta = -1$: The former assumes the same amount of selection that goes into the same direction, whereas the latter assumes the same amount of selection that goes into the opposite direction. R^2_{BM} and $R^2_{RM_{IV}}$, respectively, are the R-squared from the baseline regression model (Baseline model) and restricted regression model with only regional fixed effects controlled for (Restricted model IV) defined above. R^2_{max} is the R-squared from a regression that controls for all observed and unobserved covariates. While R^2_{max} is unobserved, we know that R^2_{max} is bounded by the upper bound 1 ($R^2_{max} = 1$) and this value gives the most conservative estimate of the genocide impacts, γ^*. R^2_{max} below 1 is considered in empirical works based on her recommendation (she derives a cutoff value of 1.3 from experimental data as a multiplier for the R-squared from restricted regression models). Given that we use non-experimental data, we consider three conservative cases: (1) $R^2_{max} = 1.5 \times R^2_{BM}$, (2) $R^2_{max} = 2.0 \times R^2_{BM}$, and (3) $R^2_{max} = 1$. In the first case ($R^2_{max} = 1.5 \times R^2_{BM}$), regardless of the direction on unobserved selection, the genocide impacts are estimated negatively only for children aged 15-21 and 6-14 of the couples who had their first child in 1977-1979 for LS II and IV. For the former children, this holds true even for the most conservative case ($R^2_{max} = 1$). These results suggest that omitted variable bias is unlikely to be significant to alter our conclusion.

References

[1] National Institute of Statistics. Cambodia Demographic and Health Survey 2000. Phnom Penh: National Institute of Statistics, Ministry of Planning, ORC Macro.; 2001.

[2] Fisher RA. Statistical Methods for Research Workers. 1st ed. London: Oliver and Boyd; 1925.

[3] Kogure K, Takasaki Y. Conflict, Institutions, and Economic Behavior: Legacies of the Cambodian Genocide; 2016. Unpublished manuscript, CIRJE-F-1034, University of Tokyo.

[4] Oster E. Unobservable Selection and Coefficient Stability: Theory and Evidence; 2018. Journal of Business & Economic Statistics (forthcoming).

[5] Altonji JG, Elder TE, Taber CR. Selection on Observed and Unobserved Vari-

ables: Assessing the Effectiveness of Catholic Schools. Journal of Political Economy. 2005;113(1):151–184.

Table A: Means and standard deviations – killing-site characteristics

Area:	Urban & Rural	Rural				
Sample:		GS	LS I	LS II	LS III	LS IV
Variable	[1]	[2]	[3]	[4]	[5]	[6]
Prop. of killing sites with info. about victims	0.642<br>(0.480)	0.664<br>(0.473)	0.667<br>(0.472)	0.722<br>(0.450)	1.000<br>(0.000)	1.000<br>(0.000)
ln (Num. of victims)	6.518<br>(1.940)	6.468<br>(1.958)	6.468<br>(1.958)	6.015<br>(1.614)	6.468<br>(1.958)	6.015<br>(1.614)
N	514	435	433	115	289	83

The unit of observation is the killing site. Standard deviations are reported in parentheses. GS–Global Sample; LS–Local Sample.

Table B: Location determinants of killing sites

Area:	Urban & Rural		Rural	
Variable	[1]	[2]	[3]	[4]
Distance to major roads (km)	-0.006***	-0.008***	-0.005***	-0.008***
	(0.001)	(0.001)	(0.001)	(0.001)
Prop. of non-migrant women aged 36-50 with grade 1-5	0.086***		0.085***	
	(0.020)		(0.020)	
Prop. of non-migrant women aged 36-50 with grade 6 or above	0.291***		0.268***	
	(0.033)		(0.035)	
Prop. of migrant households		0.039***		0.027**
		(0.013)		(0.013)
Zone and district fixed effects	√	√	√	√
N	8,247	10,503	7,903	10,022
Prop. of villages located near K.S.	0.234	0.235	0.227	0.224
R-squared	0.136	0.131	0.120	0.109

The table reports OLS estimates where the unit of observation is the village. The dependent variable is an indicator variable equal to 1 if villages are located within 3.0 km of killing sites and 0 otherwise. Robust standard errors are reported in parentheses. *** $p < 0.01$; ** $p < 0.05$; and * $p < 0.1$.

Table C: Construction of global sample

	Description of conditions
(0)	Total number of households in the 100% count 1998 Census microdata is provided.
(1)	No households live in Phnom Penh, the capital city of Cambodia.
(2)	Households include a mother aged 34-45 and the oldest child aged 16-21.
(3)	If mother's marital status is 'married,' then the households include a father.
(4)	If households include father, then the difference in age between mother and father is in the range between -3 and 20.
(5)	Mother and father* were born in Cambodia.
(6)	Mother and father* speak Khmer (Cambodian) as their mother tongue.
(7)	Mother and father* believe in Buddhism.
(8)	The highest grade of school that mother, father,* and children completed is not missing or other.
(9)	The highest grade that mother and father* completed does not exceed the one they could attain before 1975.
(10)	The information of housing (light, fuel, water, and toilet) conditions is not missing or other.
(11)	No households live in a 'special settlement.'
(12)	Number of children born alive to the mother is equal to number of children living together at the time of the 1998 Census.
(13)	If the age of ith child is the same as that of $i+1$th child, then the two children were born in the same birth district.
(14)	Mother's marital status is 'married.'
(15)	All households live in rural areas.
(16)	Both mother and father have never migrated outside their birth villages before.
(17)	Complete information about the variables used in our analysis is available.

The table shows the procedures for developing the global sample used for our analysis. The sample is developed from the 100% count 1998 Census microdata, with 2,188,177 households. Columns 2 and 3 describe conditions and the number of households that satisfy the conditions, respectively. 'father*' is used only for 'married' households. The resulting sample consists of 43,535 households

Table D: Means and standard deviations – household characteristics

A. Households with their first child born in 1977-1979						
Sample:	SP	GS	LS I	LS II	LS III	LS IV
Variable	[1]	[2]	[3]	[4]	[5]	[6]
	A-1. Parental characteristics					
Mother's age	40.609<br>(2.714)	40.595<br>(2.714)	40.565<br>(2.716)	40.647<br>(2.667)	40.578<br>(2.688)	40.621<br>(2.740)
Father's age	44.046<br>(4.632)	44.018<br>(4.617)	43.964<br>(4.511)	44.183<br>(4.521)	44.078<br>(4.593)	44.295<br>(4.589)
Mother without any grade	0.411<br>(0.492)	0.405<br>(0.491)	0.368<br>(0.482)	0.389<br>(0.480)	0.388<br>(0.488)	0.388<br>(0.488)
Mother with grade 1-5	0.479<br>(0.500)	0.485<br>(0.500)	0.513<br>(0.500)	0.518<br>(0.500)	0.510<br>(0.500)	0.510<br>(0.500)
Mother with grade 6 or above	0.110<br>(0.313)	0.110<br>(0.313)	0.120<br>(0.324)	0.121<br>(0.326)	0.100<br>(0.303)	0.102<br>(0.300)
Father without any grade	0.200<br>(0.400)	0.195<br>(0.396)	0.169<br>(0.375)	0.161<br>(0.368)	0.176<br>(0.381)	0.174<br>(0.379)
Father with grade 1-5	0.510<br>(0.500)	0.512<br>(0.500)	0.521<br>(0.500)	0.507<br>(0.500)	0.542<br>(0.498)	0.527<br>(0.500)
Father with grade 6 or above	0.290<br>(0.454)	0.293<br>(0.455)	0.310<br>(0.463)	0.332<br>(0.471)	0.283<br>(0.450)	0.300<br>(0.458)
N	11,736	11,141	5,738	2,137	2,583	1,008
	A-2. Children's educational outcomes					
	Age 15-21					
Years of schooling	4.186<br>(2.755)	4.242<br>(2.756)	4.544<br>(2.721)	4.513<br>(2.732)	4.299<br>(2.709)	4.176<br>(2.739)
N	11,736	11,141	5,738	2,137	2,583	1,008
	Age 6-14					
Grade progression	-3.665<br>(1.485)	-3.651<br>(1.480)	-3.539<br>(1.470)	-3.551<br>(1.473)	-3.617<br>(1.459)	-3.669<br>(1.464)
N	11,087	10,520	5,402	2,027	2,419	951

Continue

Table D: Means and standard deviations – household characteristics

B. Households with their first child born in 1980						
Sample:	SP	GS	LS I	LS II	LS III	LS IV
Variable	[1]	[2]	[3]	[4]	[5]	[6]
	B-1. Parental characteristics					
Mother's age	39.386	39.379	39.369	39.398	39.372	39.497
	(2.735)	(2.731)	(2.723)	(2.734)	(2.794)	(2.747)
Father's age	42.178	42.163	42.149	42.253	42.108	42.296
	(4.400)	(4.398)	(4.364)	(4.274)	(4.427)	(4.400)
Mother without any grade	0.426	0.418	0.385	0.388	0.411	0.423
	(0.495)	(0.493)	(0.487)	(0.487)	(0.492)	(0.494)
Mother with grade 1-5	0.482	0.489	0.514	0.513	0.511	0.494
	(0.500)	(0.500)	(0.500)	(0.500)	(0.500)	(0.500)
Mother with grade 6 or above	0.092	0.093	0.100	0.099	0.078	0.084
	(0.290)	(0.291)	(0.300)	(0.299)	(0.269)	(0.277)
Father without any grade	0.212	0.208	0.187	0.186	0.195	0.220
	(0.409)	(0.406)	(0.390)	(0.389)	(0.396)	(0.414)
Father with grade 1-5	0.531	0.534	0.540	0.532	0.562	0.525
	(0.499)	(0.499)	(0.498)	(0.499)	(0.496)	(0.500)
Father with grade 6 or above	0.257	0.258	0.273	0.282	0.243	0.255
	(0.437)	(0.437)	(0.445)	(0.450)	(0.429)	(0.436)
N	11,235	10,642	5,474	2,154	2,349	1,015
	B-2. Children's educational outcomes					
	Age 15-18					
Years of schooling	4.325	4.385	4.674	4.636	4.278	4.177
	(2.823)	(2.821)	(2.819)	(2.837)	(2.713)	(2.788)
N	11,235	10,642	5,474	2,154	2,349	1,015
	Age 6-14					
Grade progression	-3.620	-3.601	-3.508	-3.529	-3.601	-3.623
	(1.447)	(1.440)	(1.427)	(1.414)	(1.413)	(1.391)
N	10,747	10,185	5,246	2,068	2,250	974

Continue

Table D: Means and standard deviations – household characteristics

C. Households with their first child born in 1981-1982						
Sample:	SP	GS	LS I	LS II	LS III	LS IV
Variable	[1]	[2]	[3]	[4]	[5]	[6]
	C-1. Parental characteristics					
Mother's age	38.270<br>(2.854)	38.266<br>(2.858)	38.238<br>(2.846)	38.162<br>(2.830)	38.159<br>(2.829)	38.144<br>(2.848)
Father's age	40.816<br>(4.598)	40.794<br>(4.583)	40.790<br>(4.552)	40.738<br>(4.468)	40.704<br>(4.603)	40.732<br>(4.545)
Mother without any grade	0.463<br>(0.499)	0.453<br>(0.498)	0.420<br>(0.494)	0.418<br>(0.493)	0.444<br>(0.497)	0.452<br>(0.498)
Mother with grade 1-5	0.468<br>(0.499)	0.477<br>(0.499)	0.504<br>(0.500)	0.503<br>(0.500)	0.495<br>(0.500)	0.477<br>(0.500)
Mother with grade 6 or above	0.069<br>(0.254)	0.070<br>(0.255)	0.076<br>(0.265)	0.079<br>(0.270)	0.061<br>(0.240)	0.071<br>(0.256)
Father without any grade	0.255<br>(0.436)	0.251<br>(0.433)	0.219<br>(0.413)	0.225<br>(0.418)	0.220<br>(0.415)	0.231<br>(0.422)
Father with grade 1-5	0.543<br>(0.498)	0.546<br>(0.498)	0.561<br>(0.496)	0.548<br>(0.498)	0.574<br>(0.494)	0.546<br>(0.498)
Father with grade 6 or above	0.203<br>(0.402)	0.204<br>(0.403)	0.221<br>(0.415)	0.227<br>(0.419)	0.205<br>(0.404)	0.222<br>(0.416)
N	20,564	19,271	9,744	4,011	4,173	1,798
	C-2. Children's educational outcomes					
	Age 15-17					
Years of schooling	4.204<br>(2.873)	4.257<br>(2.875)	4.568<br>(2.873)	4.548<br>(2.898)	4.268<br>(2.790)	4.171<br>(2.819)
N	20,564	19,271	9,744	4,011	4,173	1,798
	Age 6-14					
Grade progression	-3.593<br>(1.427)	-3.571<br>(1.420)	-3.441<br>(1.403)	-3.407<br>(1.390)	-3.555<br>(1.414)	-3.548<br>(1.392)
N	19,918	18,661	9,416	3,900	4,038	1,752

The standard deviations are reported in parentheses. SP (Study Population) – households living in the districts surveyed and not surveyed by DC-Cam; GS (Global Sample) – households living in the districts surveyed by DC-Cam; LS I (Local Sample I) – households living within 6.0 km of the killing sites in the surveyed districts; LS II (Local Sample II) – households living within 6.0 km of the selected killing sites (the 6.0 km balanced spatial clusters) in the surveyed districts; LS III (Local Sample III) – households of LS I with complete victim information for all killing sites located within 6.0 km of the villages where they live; LS IV (Local Sample IV) – households of LS II with complete victim information for all killing sites located within 6.0 km of the villages where they live.

Table E: Means and standard deviations – village characteristics

Sample:	SP	GS	LS I	LS II	LS III	LS IV
Variable	[1]	[2]	[3]	[4]	[5]	[6]
A. Households with their first child born in 1977-1979						
Distance to major roads (km)	9.952	8.829	8.466	8.748	10.785	8.936
	(12.391)	(9.295)	(9.285)	(9.870)	(9.807)	(8.814)
Prop. of non-migrant women	0.484	0.475	0.448	0.452	0.471	0.476
aged 36-50 without any grade	(0.229)	(0.227)	(0.221)	(0.226)	(0.226)	(0.226)
Prop. of non-migrant women	0.414	0.422	0.442	0.438	0.437	0.430
aged 36-50 with grade 1-5	(0.207)	(0.206)	(0.201)	(0.208)	(0.208)	(0.210)
Prop. of non-migrant women	0.102	0.103	0.109	0.111	0.092	0.095
aged 36-50 with grade 6 or above	(0.116)	(0.115)	(0.115)	(0.119)	(0.101)	(0.101)
N	4,286	3,989	1,979	839	867	376
B. Households with their first child born in 1980						
Distance to major roads (km)	9.559	8.660	8.419	8.861	10.871	9.577
	(11.569)	(9.177)	(9.234)	(9.701)	(9.866)	(9.138)
Prop. of non-migrant women	0.480	0.472	0.444	0.448	0.470	0.478
aged 36-50 without any grade	(0.228)	(0.226)	(0.221)	(0.226)	(0.229)	(0.227)
Prop. of non-migrant women	0.416	0.424	0.443	0.438	0.438	0.427
aged 36-50 with grade 1-5	(0.207)	(0.206)	(0.202)	(0.209)	(0.210)	(0.211)
Prop. of non-migrant women	0.104	0.104	0.112	0.114	0.091	0.094
aged 36-50 with grade 6 or above	(0.118)	(0.117)	(0.118)	(0.121)	(0.103)	(0.104)
N	4,448	4,154	2,098	889	926	403
C. Households with their first child born in 1981-1982						
Distance to major roads (km)	9.731	8.686	8.422	8.595	10.831	9.466
	(11.903)	(9.075)	(9.123)	(9.495)	(9.516)	(8.684)
Prop. of non-migrant women	0.492	0.482	0.456	0.457	0.479	0.483
aged 36-50 without any grade	(0.233)	(0.230)	(0.222)	(0.222)	(0.226)	(0.222)
Prop. of non-migrant women	0.408	0.417	0.436	0.433	0.431	0.427
aged 36-50 with grade 1-5	(0.211)	(0.209)	(0.205)	(0.208)	(0.210)	(0.209)
Prop. of non-migrant women	0.100	0.101	0.108	0.110	0.090	0.091
aged 36-50 with grade 6 or above	(0.118)	(0.117)	(0.117)	(0.119)	(0.108)	(0.110)
N	5,595	5,197	2,577	1,128	1,137	512

See the notes to Table D.

Table F: Treatment-control mean differences – household characteristics

A. Households with their first child born in 1977-1979									
Sample:	Global Sample			Local Sample I (All spatial clusters)			Local Sample II (Balanced spatial clusters)		
	< 3 km of K.S.	≥ 3 km of K.S.	diff.	< 3 km of K.S.	≥ 3 km of K.S.	diff.	< 3 km of K.S.	≥ 3 km of K.S.	diff.
Variable	[1]	[2]	[3]	[4]	[5]	[6]	[7]	[8]	[9]
	A-1. Parental characteristics								
Mother's age	40.602<br>(2.692)	40.593<br>(2.722)	0.009<br>(0.059)	40.548<br>(2.713)	40.579<br>(2.720)	-0.030<br>(0.072)	40.625<br>(2.670)	40.666<br>(2.666)	-0.041<br>(0.116)
Father's age	44.016<br>(4.491)	44.019<br>(4.660)	-0.003<br>(0.100)	43.916<br>(4.482)	44.004<br>(4.535)	-0.087<br>(0.120)	44.131<br>(4.576)	44.226<br>(4.477)	-0.096<br>(0.197)
Mother without any grade	0.339<br>(0.474)	0.428<br>(0.495)	-0.089***<br>(0.011)	0.340<br>(0.474)	0.391<br>(0.488)	-0.050***<br>(0.013)	0.315<br>(0.465)	0.398<br>(0.490)	-0.083***<br>(0.021)
Mother with grade 1-5	0.527<br>(0.499)	0.470<br>(0.499)	0.057***<br>(0.011)	0.529<br>(0.499)	0.499<br>(0.500)	0.030**<br>(0.013)	0.562<br>(0.496)	0.482<br>(0.500)	0.080***<br>(0.022)
Mother with grade 6 or above	0.134<br>(0.340)	0.102<br>(0.303)	0.032***<br>(0.007)	0.131<br>(0.337)	0.110<br>(0.313)	0.021**<br>(0.009)	0.123<br>(0.329)	0.119<br>(0.324)	0.004<br>(0.014)
Father without any grade	0.156<br>(0.363)	0.209<br>(0.407)	-0.053***<br>(0.009)	0.160<br>(0.366)	0.177<br>(0.382)	-0.017*<br>(0.010)	0.156<br>(0.363)	0.165<br>(0.371)	-0.008<br>(0.016)
Father with grade 1-5	0.512<br>(0.500)	0.512<br>(0.500)	0.000<br>(0.011)	0.511<br>(0.500)	0.529<br>(0.499)	-0.017<br>(0.013)	0.508<br>(0.500)	0.507<br>(0.500)	0.001<br>(0.022)
Father with grade 6 or above	0.332<br>(0.471)	0.279<br>(0.448)	0.054***<br>(0.010)	0.329<br>(0.470)	0.295<br>(0.456)	0.034***<br>(0.012)	0.336<br>(0.472)	0.328<br>(0.470)	0.007<br>(0.020)
N	2,886	8,255		2,632	3,106		965	1,172	
	A-2. Children's educational outcomes								
	Age 15-21								
Years of schooling	4.826<br>(2.754)	4.038<br>(2.728)	0.788***<br>(0.059)	4.780<br>(2.758)	4.343<br>(2.675)	0.437***<br>(0.072)	4.512<br>(2.759)	4.515<br>(2.711)	-0.003<br>(0.119)
N	2,886	8,255		2,632	3,106		965	1,172	
	Age 6-14								
Grade progression	-3.473<br>(1.428)	-3.712<br>(1.492)	0.240***<br>(0.033)	-3.480<br>(1.441)	-3.588<br>(1.493)	0.108***<br>(0.040)	-3.556<br>(1.429)	-3.547<br>(1.509)	-0.009<br>(0.066)
N	2,697	7,823		2,460	2,942		908	1,119	

Continue

Table F: Treatment-control mean differences – household characteristics

B. Households with their first child born in 1980									
Sample:	Global Sample			Local Sample I (All spatial clusters)			Local Sample II (Balanced spatial clusters)		
	< 3 km of K.S.	≥ 3 km of K.S.	diff.	< 3 km of K.S.	≥ 3 km of K.S.	diff.	< 3 km of K.S.	≥ 3 km of K.S.	diff.
Variable	[1]	[2]	[3]	[4]	[5]	[6]	[7]	[8]	[9]
	B-1. Parental characteristics								
Mother's age	39.351<br>(2.750)	39.388<br>(2.725)	-0.037<br>(0.062)	39.323<br>(2.741)	39.406<br>(2.709)	-0.083<br>(0.074)	39.286<br>(2.795)	39.486<br>(2.684)	-0.200*<br>(0.119)
Father's age	42.236<br>(4.400)	42.139<br>(4.398)	0.097<br>(0.099)	42.155<br>(4.352)	42.143<br>(4.374)	0.012<br>(0.119)	42.196<br>(4.198)	42.297<br>(4.334)	-0.101<br>(0.186)
Mother without any grade	0.357<br>(0.479)	0.438<br>(0.496)	-0.080***<br>(0.011)	0.360<br>(0.480)	0.405<br>(0.491)	-0.046***<br>(0.013)	0.372<br>(0.484)	0.400<br>(0.490)	-0.028<br>(0.021)
Mother with grade 1-5	0.533<br>(0.499)	0.475<br>(0.499)	0.058***<br>(0.011)	0.535<br>(0.499)	0.498<br>(0.500)	0.037***<br>(0.014)	0.544<br>(0.498)	0.489<br>(0.500)	0.055**<br>(0.022)
Mother with grade 6 or above	0.110<br>(0.313)	0.088<br>(0.283)	0.022***<br>(0.007)	0.105<br>(0.306)	0.097<br>(0.295)	0.008<br>(0.008)	0.084<br>(0.277)	0.111<br>(0.314)	-0.027**<br>(0.013)
Father without any grade	0.176<br>(0.381)	0.218<br>(0.413)	-0.042***<br>(0.009)	0.183<br>(0.387)	0.190<br>(0.393)	-0.007<br>(0.011)	0.186<br>(0.390)	0.186<br>(0.389)	0.000<br>(0.017)
Father with grade 1-5	0.531<br>(0.499)	0.536<br>(0.499)	-0.004<br>(0.011)	0.530<br>(0.499)	0.548<br>(0.498)	-0.019<br>(0.014)	0.531<br>(0.499)	0.533<br>(0.499)	-0.001<br>(0.022)
Father with grade 6 or above	0.292<br>(0.455)	0.247<br>(0.431)	0.046***<br>(0.010)	0.287<br>(0.453)	0.261<br>(0.439)	0.026**<br>(0.012)	0.283<br>(0.450)	0.281<br>(0.450)	0.001<br>(0.020)
N	2,602	8,040		2,398	3,076		945	1,209	
	B-2. Children's educational outcomes								
	Age 15-18								
Years of schooling	4.904<br>(2.852)	4.218<br>(2.791)	0.687***<br>(0.063)	4.863<br>(2.865)	4.527<br>(2.775)	0.336***<br>(0.077)	4.669<br>(2.822)	4.610<br>(2.848)	0.059<br>(0.123)
N	2,602	8,040		2,398	3,076		945	1,209	
	Age 6-14								
Grade progression	-3.430<br>(1.422)	-3.656<br>(1.442)	0.226***<br>(0.033)	-3.450<br>(1.421)	-3.554<br>(1.430)	0.104***<br>(0.040)	-3.482<br>(1.367)	-3.565<br>(1.450)	0.083<br>(0.063)
N	2,490	7,695		2,294	2,952		909	1,159	

Continue

Table F: Treatment-control mean differences – household characteristics

C. Households with their first child born in 1981-1982									
Sample:	Global Sample			Local Sample I (All spatial clusters)			Local Sample II (Balanced spatial clusters)		
	< 3 km of K.S.	≥ 3 km of K.S.	diff.	< 3 km of K.S.	≥ 3 km of K.S.	diff.	< 3 km of K.S.	≥ 3 km of K.S.	diff.
Variable	[1]	[2]	[3]	[4]	[5]	[6]	[7]	[8]	[9]
	C-1. Parental characteristics								
Mother's age	38.293<br>(2.837)	38.257<br>(2.865)	0.037<br>(0.048)	38.266<br>(2.831)	38.216<br>(2.857)	0.049<br>(0.058)	38.090<br>(2.813)	38.219<br>(2.843)	-0.130<br>(0.090)
Father's age	40.887<br>(4.506)	40.765<br>(4.607)	0.122<br>(0.077)	40.862<br>(4.517)	40.733<br>(4.579)	0.130<br>(0.093)	40.738<br>(4.484)	40.738<br>(4.456)	0.000<br>(0.142)
Mother without any grade	0.396<br>(0.489)	0.471<br>(0.499)	-0.076***<br>(0.008)	0.400<br>(0.490)	0.435<br>(0.496)	-0.035***<br>(0.010)	0.390<br>(0.488)	0.439<br>(0.496)	-0.049***<br>(0.016)
Mother with grade 1-5	0.515<br>(0.500)	0.465<br>(0.499)	0.049***<br>(0.008)	0.515<br>(0.500)	0.496<br>(0.500)	0.019*<br>(0.010)	0.535<br>(0.499)	0.478<br>(0.500)	0.056***<br>(0.016)
Mother with grade 6 or above	0.090<br>(0.286)	0.064<br>(0.244)	0.026***<br>(0.004)	0.085<br>(0.279)	0.069<br>(0.254)	0.015***<br>(0.005)	0.075<br>(0.264)	0.082<br>(0.275)	-0.007<br>(0.009)
Father without any grade	0.207<br>(0.405)	0.264<br>(0.441)	-0.057***<br>(0.007)	0.208<br>(0.406)	0.227<br>(0.419)	-0.018**<br>(0.008)	0.223<br>(0.416)	0.227<br>(0.419)	-0.004<br>(0.013)
Father with grade 1-5	0.552<br>(0.497)	0.544<br>(0.498)	0.008<br>(0.008)	0.558<br>(0.497)	0.563<br>(0.496)	-0.005<br>(0.010)	0.559<br>(0.497)	0.539<br>(0.499)	0.020<br>(0.016)
Father with grade 6 or above	0.241<br>(0.428)	0.192<br>(0.394)	0.049***<br>(0.007)	0.234<br>(0.423)	0.210<br>(0.407)	0.024***<br>(0.008)	0.218<br>(0.413)	0.234<br>(0.424)	-0.016<br>(0.013)
N	4,654	14,617		4,316	5,428		1,764	2,247	
	C-2. Children's educational outcomes								
	Age 15-17								
Years of schooling	4.831<br>(2.935)	4.075<br>(2.832)	0.756***<br>(0.048)	4.775<br>(2.940)	4.403<br>(2.808)	0.372***<br>(0.058)	4.572<br>(2.937)	4.530<br>(2.868)	0.042<br>(0.092)
N	4,654	14,617		4,316	5,428		1,764	2,247	
	Age 6-14								
Grade progression	-3.362<br>(1.427)	-3.637<br>(1.412)	0.274***<br>(0.024)	-3.380<br>(1.423)	-3.488<br>(1.386)	0.109***<br>(0.029)	-3.364<br>(1.395)	-3.441<br>(1.385)	0.077*<br>(0.045)
N	4,476	14,185		4,149	5,267		1,705	2,195	

The unit of observation is the household. Standard deviations are reported in parentheses in columns 1, 2, 4, 5, 7, and 8. Standard errors of differences in means are reported in parentheses in columns 3, 6, and 9. *** $p < 0.01$; ** $p < 0.05$; and * $p < 0.1$. p-values for the difference in the means are based on two-sided unpaired t-tests.

Table G: Treatment-control mean differences – village characteristics

Sample:	Global Sample			Local Sample I (All spatial clusters)			Local Sample II (Balanced spatial clusters)		
	< 3 km of K.S.	≥ 3 km of K.S.	diff.	< 3 km of K.S.	≥ 3 km of K.S.	diff.	< 3 km of K.S.	≥ 3 km of K.S.	diff.
Variable	[1]	[2]	[3]	[4]	[5]	[6]	[7]	[8]	[9]
	A. Households with their first child born in 1977-1979								
Distance to major roads (km)	8.153	9.041	-0.889**	8.390	8.527	-0.137	8.766	8.732	0.034
	(8.852)	(9.421)	(0.345)	(9.035)	(9.482)	(0.420)	(10.023)	(9.746)	(0.684)
Prop. of non-migrant women aged 36-50 without any grade	0.417	0.494	-0.077***	0.422	0.469	-0.047***	0.425	0.475	-0.049***
	(0.218)	(0.226)	(0.008)	(0.217)	(0.221)	(0.010)	(0.224)	(0.226)	(0.016)
Prop. of non-migrant women aged 36-50 with grade 1-5	0.454	0.411	0.043***	0.453	0.434	0.020**	0.456	0.422	0.035**
	(0.195)	(0.208)	(0.008)	(0.197)	(0.203)	(0.009)	(0.210)	(0.204)	(0.014)
Prop. of non-migrant women aged 36-50 with grade 6 or above	0.129	0.095	0.034***	0.125	0.097	0.028***	0.118	0.104	0.014*
	(0.127)	(0.109)	(0.004)	(0.125)	(0.105)	(0.005)	(0.131)	(0.107)	(0.008)
N	953	3,036		879	1,100		390	449	
	B. Households with their first child born in 1980								
Distance to major roads (km)	8.277	8.775	-0.499	8.519	8.346	0.173	9.183	8.602	0.581
	(8.931)	(9.249)	(0.337)	(9.098)	(9.338)	(0.408)	(9.975)	(9.477)	(0.655)
Prop. of non-migrant women aged 36-50 without any grade	0.407	0.491	-0.085***	0.412	0.468	-0.057***	0.416	0.474	-0.058***
	(0.216)	(0.226)	(0.008)	(0.215)	(0.222)	(0.010)	(0.224)	(0.225)	(0.015)
Prop. of non-migrant women aged 36-50 with grade 1-5	0.464	0.412	0.052***	0.463	0.429	0.034***	0.464	0.416	0.048***
	(0.197)	(0.207)	(0.008)	(0.199)	(0.203)	(0.009)	(0.213)	(0.203)	(0.014)
Prop. of non-migrant women aged 36-50 with grade 6 or above	0.130	0.097	0.033***	0.125	0.103	0.022***	0.120	0.110	0.010
	(0.124)	(0.114)	(0.004)	(0.123)	(0.113)	(0.005)	(0.124)	(0.118)	(0.008)
N	964	3,190		895	1,203		397	492	
	C. Households with their first child born in 1981-1982								
Distance to major roads (km)	8.160	8.843	-0.683**	8.411	8.430	-0.019	8.813	8.732	0.394
	(8.851)	(9.136)	(0.299)	(9.016)	(9.205)	(0.363)	(9.896)	(9.746)	(0.569)
Prop. of non-migrant women aged 36-50 without any grade	0.432	0.497	-0.066***	0.437	0.470	-0.033***	0.440	0.475	-0.032**
	(0.223)	(0.230)	(0.008)	(0.222)	(0.221)	(0.009)	(0.228)	(0.226)	(0.013)
Prop. of non-migrant women aged 36-50 with grade 1-5	0.445	0.409	0.035***	0.444	0.430	0.013*	0.446	0.422	0.024*
	(0.201)	(0.211)	(0.007)	(0.203)	(0.206)	(0.008)	(0.215)	(0.204)	(0.012)
Prop. of non-migrant women aged 36-50 with grade 6 or above	0.124	0.094	0.030***	0.119	0.099	0.020***	0.115	0.104	0.008
	(0.124)	(0.113)	(0.004)	(0.121)	(0.112)	(0.005)	(0.121)	(0.107)	(0.007)
N	1,195	4,002		1,107	1,470		503	625	

The unit of observation is the village. Standard deviations are reported in parentheses in columns 1, 2, 4, 5, 7, and 8. Standard errors of differences in means are reported in parentheses in columns 3, 6, and 9. *** $p < 0.01$; ** $p < 0.05$; and * $p < 0.1$. p-values for the difference in the means are based on two-sided unpaired t-tests.

Table H: External validity – mean differences across samples

A. Households with their first child born in 1977-1979									
Reference sample:	SP					GS	LS I	LS I	LS II
Local sample:	vs. GS	vs. LS I	vs. LS II	vs. LS III	vs. LS IV	vs. LS I	vs. LS II	vs. LS III	vs. LS IV
Variable	[1]	[2]	[3]	[4]	[5]	[6]	[7]	[8]	[9]
	A-1. Parental characteristics								
Mother's age	0.000	0.000	-0.001	0.000	-0.001	-0.001	-0.002	0.002	0.002
	(0.001)	(0.002)	(0.002)	(0.002)	(0.001)	(0.002)	(0.002)	(0.002)	(0.003)
Father's age	0.000	0.000	0.001	0.002**	0.002**	0.001	0.000	0.001	0.002
	(0.001)	(0.001)	(0.001)	(0.001)	(0.001)	(0.001)	(0.001)	(0.001)	(0.002)
Mother with grade 1-5	0.007	0.047***	0.022***	-0.001	0.002	0.037***	0.008	-0.036***	-0.011
	(0.004)	(0.010)	(0.008)	(0.009)	(0.006)	(0.010)	(0.011)	(0.012)	(0.017)
Mother with grade 6 or above	0.001	0.058***	0.017	-0.015	-0.006	0.056***	-0.002	-0.047***	-0.037
	(0.007)	(0.017)	(0.013)	(0.013)	(0.009)	(0.015)	(0.017)	(0.016)	(0.025)
Father with grade 1-5	0.012*	0.049***	0.016*	0.019*	0.005	0.039***	0.006	-0.032**	-0.002
	(0.006)	(0.013)	(0.009)	(0.010)	(0.007)	(0.012)	(0.013)	(0.015)	(0.022)
Father with grade 6 or above	0.012*	0.068***	0.040***	0.007	0.009	0.069***	0.018	-0.049***	-0.011
	(0.007)	(0.014)	(0.011)	(0.012)	(0.008)	(0.014)	(0.015)	(0.016)	(0.023)
Zone fixed effects	√	√	√	√	√	√	√	√	√
District fixed effects						√	√	√	√
N	11,736	11,736	11,736	11,736	11,736	11,141	5,738	5,738	2,137
N (Local sample)	11,141	5,738	2,137	2,583	1,008	5,738	2,137	2,583	1,008
R-squared	0.120	0.054	0.020	0.051	0.016	0.236	0.506	0.481	0.577
p-value of the listed variables	0.095	0.000	0.000	0.040	0.162	0.000	0.770	0.000	0.538
	A-2. Village characteristics								
Distance to major roads (km)	-0.006***	-0.004***	-0.001	0.001	-0.001***	-0.005***	0.014***	0.015***	0.022***
	0.000	(0.001)	(0.000)	(0.000)	(0.000)	(0.001)	(0.002)	(0.003)	(0.006)
Proportion of non-migrant women aged 36-50 with grade 1-5	0.068***	0.232***	0.078**	0.019	-0.010	0.132***	0.025	-0.220***	-0.013
	(0.018)	(0.037)	(0.030)	(0.031)	(0.022)	(0.042)	(0.052)	(0.050)	(0.077)
Proportion of non-migrant women aged 36-50 with grade 6 or above	-0.033	0.224***	0.123**	-0.145***	-0.048	0.288***	0.136*	-0.241***	-0.075
	(0.035)	(0.066)	(0.053)	(0.048)	(0.034)	(0.069)	(0.082)	(0.079)	(0.127)
Zone fixed effects	√	√	√	√	√	√	√	√	√
District fixed effects						√	√	√	√
N	4,286	4,286	4,286	4,286	4,286	3,989	1,979	1,979	839
N (Local sample)	3,989	1,979	839	867	376	1,979	839	867	376
R-squared	0.217	0.057	0.019	0.048	0.015	0.209	0.502	0.486	0.558
p-value of the listed variables	0.000	0.000	0.003	0.006	0.021	0.000	0.000	0.000	0.002

Continue

Table H: External validity – mean differences across samples

B. Households with their first child born in 1980									
Reference sample:	SP					GS	LS I	LS I	LS II
Local sample:	vs. GS	vs. LS I	vs. LS II	vs. LS III	vs. LS IV	vs. LS I	vs. LS II	vs. LS III	vs. LS IV
Variable	[1]	[2]	[3]	[4]	[5]	[6]	[7]	[8]	[9]
	B-1. Parental characteristics								
Mother's age	0.000	0.001	-0.001	0.003	0.002	0.001	-0.001	0.002	0.001
	(0.001)	(0.002)	(0.002)	(0.002)	(0.001)	(0.002)	(0.002)	(0.002)	(0.003)
Father's age	0.000	0.000	0.001	0.000	0.000	0.000	0.001	0.001	0.004*
	(0.001)	(0.001)	(0.001)	(0.001)	(0.001)	(0.001)	(0.001)	(0.001)	(0.002)
Mother with grade 1-5	0.012***	0.043***	0.013	-0.012	-0.004	0.017	0.000	-0.034***	-0.004
	(0.005)	(0.011)	(0.008)	(0.009)	(0.006)	(0.010)	(0.012)	(0.011)	(0.017)
Mother with grade 6 or above	0.011	0.052***	0.011	-0.034**	-0.011	0.026	-0.006	-0.031*	0.002
	(0.008)	(0.018)	(0.015)	(0.014)	(0.010)	(0.017)	(0.020)	(0.018)	(0.026)
Father with grade 1-5	0.009	0.032**	0.006	0.013	-0.012	0.017	0.005	-0.008	-0.017
	(0.006)	(0.013)	(0.010)	(0.010)	(0.007)	(0.012)	(0.014)	(0.014)	(0.021)
Father with grade 6 or above	0.005	0.056***	0.023**	0.001	-0.008	0.046***	0.023	-0.040**	-0.068***
	(0.007)	(0.015)	(0.012)	(0.012)	(0.009)	(0.014)	(0.016)	(0.016)	(0.024)
Zone fixed effects	√	√	√	√	√	√	√	√	√
District fixed effects						√	√	√	√
N	11,235	11,235	11,235	11,235	11,235	10,642	5,474	5,474	2,154
N (Local sample)	10,642	5,474	2,154	2,349	1,015	5,474	2,154	2,349	1,015
R-squared	0.118	0.045	0.020	0.055	0.017	0.195	0.453	0.518	0.601
p-value of the listed variables	0.019	0.000	0.067	0.066	0.205	0.001	0.771	0.000	0.021
	B-2. Village characteristics								
Distance to major roads (km)	-0.006***	-0.004***	0.000	0.001**	0.000	-0.002	0.015***	0.013***	0.022***
	0.000	(0.001)	(0.001)	(0.001)	(0.000)	(0.001)	(0.002)	(0.003)	(0.006)
Proportion of non-migrant women aged 36-50 with grade 1-5	0.080***	0.239***	0.065**	0.017	-0.016	0.143***	0.053	-0.240***	-0.080
	(0.017)	(0.036)	(0.030)	(0.030)	(0.022)	(0.041)	(0.050)	(0.048)	(0.073)
Proportion of non-migrant women aged 36-50 with grade 6 or above	-0.038	0.275***	0.149***	-0.169***	-0.057*	0.363***	0.054	-0.275***	-0.122
	(0.033)	(0.064)	(0.051)	(0.047)	(0.033)	(0.066)	(0.079)	(0.079)	(0.119)
Zone fixed effects	√	√	√	√	√	√	√	√	√
District fixed effects						√	√	√	√
N	4,448	4,448	4,448	4,448	4,448	4,154	2,098	2,098	889
N (Local sample)	4,154	2,098	889	926	403	2,098	889	926	403
R-squared	0.192	0.059	0.023	0.057	0.015	0.198	0.474	0.497	0.575
p-value of the listed variables	0.000	0.000	0.007	0.000	0.192	0.000	0.000	0.000	0.001

Continue

Table H: External validity – mean differences across samples

C. Households with their first child born in 1981-1982									
Reference sample:	SP					GS	LS I	LS I	LS II
Local sample:	vs. GS	vs. LS I	vs. LS II	vs. LS III	vs. LS IV	vs. LS I	vs. LS II	vs. LS III	vs. LS IV
Variable	[1]	[2]	[3]	[4]	[5]	[6]	[7]	[8]	[9]
	C-1. Parental characteristics								
Mother's age	0.000<br>(0.001)	-0.002<br>(0.002)	-0.004***<br>(0.001)	-0.002<br>(0.001)	-0.002*<br>(0.001)	-0.002<br>(0.001)	-0.005***<br>(0.002)	0.001<br>(0.002)	0.003<br>(0.002)
Father's age	0.000<br>(0.000)	0.000<br>(0.001)	0.001<br>(0.001)	0.000<br>(0.001)	0.000<br>(0.001)	0.000<br>(0.001)	0.001<br>(0.001)	0.000<br>(0.001)	-0.001<br>(0.002)
Mother with grade 1-5	0.021***<br>(0.004)	0.042***<br>(0.008)	0.020***<br>(0.006)	-0.010<br>(0.006)	-0.005<br>(0.004)	0.012<br>(0.007)	0.022***<br>(0.008)	-0.027***<br>(0.008)	-0.005<br>(0.013)
Mother with grade 6 or above	0.022***<br>(0.007)	0.055***<br>(0.015)	0.032**<br>(0.013)	-0.031***<br>(0.012)	-0.004<br>(0.009)	0.028**<br>(0.014)	0.033*<br>(0.017)	-0.036**<br>(0.015)	-0.015<br>(0.023)
Father with grade 1-5	0.000<br>(0.004)	0.053***<br>(0.009)	0.009<br>(0.007)	0.027***<br>(0.007)	0.004<br>(0.005)	0.044***<br>(0.009)	-0.010<br>(0.010)	-0.008<br>(0.010)	-0.009<br>(0.015)
Father with grade 6 or above	-0.004<br>(0.006)	0.080***<br>(0.011)	0.027***<br>(0.009)	0.029***<br>(0.009)	0.015**<br>(0.007)	0.077***<br>(0.011)	-0.005<br>(0.013)	-0.010<br>(0.012)	0.014<br>(0.018)
Zone fixed effects	√	√	√	√	√	√	√	√	√
District fixed effects						√	√	√	√
N	20,564	20,564	20,564	20,564	20,564	19,271	9,744	9,744	4,011
N (Local sample)	19,271	9,744	4,011	4,173	1,798	9,744	4,011	4,173	1,798
R-squared	0.118	0.042	0.016	0.048	0.013	0.201	0.471	0.510	0.547
p-value of the listed variables	0.000	0.000	0.000	0.001	0.133	0.000	0.007	0.006	0.627
	C-2. Village characteristics								
Distance to major roads (km)	-0.006***<br>(0.000)	-0.004***<br>(0.001)	0.000<br>(0.000)	0.001**<br>(0.000)	0.000<br>(0.000)	-0.004***<br>(0.001)	0.014***<br>(0.002)	0.014***<br>(0.002)	0.021***<br>(0.005)
Proportion of non-migrant women aged 36-50 with grade 1-5	0.082***<br>(0.015)	0.219***<br>(0.032)	0.078***<br>(0.026)	0.030<br>(0.026)	0.000<br>(0.019)	0.098***<br>(0.036)	0.031<br>(0.043)	-0.173***<br>(0.043)	-0.024<br>(0.063)
Proportion of non-migrant women aged 36-50 with grade 6 or above	-0.027<br>(0.030)	0.235***<br>(0.057)	0.137***<br>(0.045)	-0.134***<br>(0.043)	-0.059*<br>(0.031)	0.273***<br>(0.059)	0.064<br>(0.076)	-0.184**<br>(0.072)	-0.034<br>(0.113)
Zone fixed effects	√	√	√	√	√	√	√	√	√
District fixed effects						√	√	√	√
N	5,595	5,595	5,595	5,595	5,595	5,197	2,577	2,577	1,128
N (Local sample)	5,197	2,577	1,128	1,137	512	2,577	1,128	1,137	512
R-squared	0.208	0.051	0.022	0.051	0.014	0.197	0.474	0.496	0.564
p-value of the listed variables	0.000	0.000	0.000	0.000	0.116	0.000	0.000	0.000	0.000

The table reports OLS estimates where the unit of observation is the household in panels A-1, B-1,and C-1 and the village in panels A-2, B-2, and C-2. The dependent variable is an indicator variable equal to 1 if households/villages are included in both the reference and local samples and 0 otherwise. Robust standard errors are reported in parentheses. The regressions in columns 1-5 do not include district fixed effects because about 60 districts in the SP (Study Population) (districts with white background, not surveyed by DC-Cam) are not included in GS (Global Sample), LSs (Local Samples) I and II (see S2 Fig). p-values are from F-tests for the joint significance of the listed variables. *** $p < 0.01$; ** $p < 0.05$; and * $p < 0.1$.

Table I: Exogeneity of the timing of childbearing – difference in number of children across subsamples

Sample:	LS II	LS IV
Dependent variable:	Num. of children	Num. of children
Variable	[1]	[2]
HH with first child born in 1977-79	0.642***	0.626***
	(0.050)	(0.071)
HH with first child born in 1980	0.359***	0.315***
	(0.042)	(0.057)
Parental characteristics	√	√
Zone, district, and spatial cluster fixed effects	√	√
N	8,302	3,821
N (HH with first child born in 1977-79)	2,137	1,008
N (HH with first child born in 1980)	2,154	1,015
R-squared	0.107	0.121
p-value of the two listed variables	0.000	0.000

The table reports OLS estimates where the unit of observation is the household. The dependent variable is the number of children in households. "HH with first child born in 1977-1979 (1980)" is an indicator variable equal to 1 if households had their first child born in 1977-1979 (1980) and 0 otherwise ("HH with first child born in 1981-1982" is excluded as the base case). Parental characteristics include the mother's and father's age and education. Robust standard errors, adjusted for clustering by village, are reported in parentheses. p-values are from F-tests for the joint significance of the two listed variables. *** $p < 0.01$; ** $p < 0.05$; and * $p < 0.1$.

Table J: Exogeneity of locations of killing sites/binary genocide measure – other subsamples

Sample:	Global Sample		Local Sample I		Local Sample II	
Subsample:	1980	1981-82	1980	1981-82	1980	1981-82
Variable	[1]	[2]	[3]	[4]	[5]	[6]
	A. Village characteristics					
Distance to major roads (km)	-0.002<br>(0.001)	-0.003***<br>(0.001)	0.001<br>(0.006)	0.000<br>(0.005)	0.000<br>(0.011)	-0.003<br>(0.010)
Prop. of non-migrant women aged 36-50 with grade 1-5	0.176***<br>(0.034)	0.074**<br>(0.030)	0.207***<br>(0.071)	0.014<br>(0.060)	0.190<br>(0.116)	0.076<br>(0.090)
Prop. of non-migrant women aged 36-50 with grade 6 or above	0.467***<br>(0.061)	0.369***<br>(0.054)	0.507***<br>(0.110)	0.350***<br>(0.095)	0.221<br>(0.180)	0.150<br>(0.144)
Zone and district fixed effects	√	√	√	√	√	√
Spatial cluster fixed effects			√	√	√	√
N	4,154	5,197	2,098	2,577	889	1,128
R-squared	0.126	0.125	0.351	0.329	0.439	0.443
p-value of the listed variables	0.000	0.000	0.000	0.003	0.285	0.575
	B. Parental characteristics					
Mother's age	-0.002<br>(0.002)	-0.001<br>(0.001)	-0.007***<br>(0.003)	-0.001<br>(0.002)	-0.003<br>(0.004)	-0.005*<br>(0.003)
Father's age	0.001<br>(0.001)	0.000<br>(0.001)	0.003*<br>(0.002)	0.000<br>(0.001)	0.003<br>(0.003)	0.001<br>(0.002)
Mother with grade 1-5	0.031***<br>(0.010)	0.020***<br>(0.008)	0.037**<br>(0.014)	0.008<br>(0.011)	0.033<br>(0.022)	0.021<br>(0.016)
Mother with grade 6 or above	0.039**<br>(0.018)	0.052***<br>(0.015)	0.028<br>(0.024)	0.052***<br>(0.019)	-0.031<br>(0.035)	-0.005<br>(0.029)
Father with grade 1-5	0.001<br>(0.011)	0.020**<br>(0.008)	-0.014<br>(0.018)	0.013<br>(0.012)	0.010<br>(0.029)	0.010<br>(0.018)
Father with grade 6 or above	0.040***<br>(0.014)	0.059***<br>(0.011)	0.029<br>(0.020)	0.036**<br>(0.015)	0.030<br>(0.031)	0.010<br>(0.023)
Zone and district fixed effects	√	√	√	√	√	√
Spatial cluster fixed effects			√	√	√	√
N	10,642	19,271	5,474	9,744	2,154	4,011
R-squared	0.128	0.134	0.370	0.349	0.463	0.452
p-value of the listed variables	0.000	0.000	0.002	0.014	0.280	0.319
Num. of killing sites	435	435	433	433	115	115

The table reports OLS estimates where the unit of observation is the village in panel A and the household in panel B. Robust standard errors are reported in parentheses in panel A and robust standard errors, adjusted for clustering by village, are reported in parentheses in panel B. See the text for description of the samples. The dependent variable is an indicator variable equal to 1 if villages are located within 3.0 km of killing sites and 0 otherwise. p-values are from F-tests for the joint significance of the listed variables. *** $p < 0.01$; ** $p < 0.05$; and * $p < 0.1$.

Table K: Construction of sample for Fisher's exact tests

	Description of conditions
(0)	Total number of households in the 100% count 1998 Census microdata is provided.
(1)	No households live in Phnom Penh, the capital city of Cambodia.
(2)	Households include a mother aged 34-45 and the oldest child aged 16-21.
(3)	If mother's marital status is 'married,' then the households include a father.
(4)	If households include father, then the difference in age between mother and father is in the range between -3 and 20.
(5)	Mother and father* were born in Cambodia.
(6)	Mother and father* speak Khmer (Cambodian) as their mother tongue.
(7)	Mother and father* believe in Buddhism.
(8)	No households live in a 'special settlement.'
(9)	Number of children born alive to the mother is equal to number of children living together at the time of the 1998 Census.
(10)	If the age of ith child is the same as that of $i+1$th child, then the two children were born in the same birth district.
(11)	Mother's marital status is 'married.'
(12)	No households include grandfather, grandmother, grandchild, other relatives, or non-relatives.
(13)	All children in households speak Khmer (Cambodian) as their mother tongue.
(14)	All children in households believe in Buddhism.
(15)	All children in households have never married.

The table shows the procedures for constructing the sample used for Fisher's exact tests. See the notes to Table C. The resulting sample consists of 108,518 households.

Table L: Killing-site characteristics with balanced spatial clusters and information about victims

Killing site:	Killing sites used for F.E. tests			Killing sites with balanced spatial clusters (B.S.C)	
Dependent variable:	Have B.S.C.	Have Victim Information	ln (Num. of Victim)	Have Victim Information	ln (Num. of Victim)
Variable	[1]	[2]	[3]	[4]	[5]
Distance to major roads (km)	0.007***	0.003	-0.027**	-0.004	-0.044*
	(0.003)	(0.003)	(0.013)	(0.005)	(0.023)
Prop. of non-migrant women aged 36-50 with grade 1-5	0.084	0.146	1.080	-0.350	1.735
	(0.137)	(0.148)	(0.753)	(0.265)	(1.185)
Prop. of non-migrant women aged 36-50 with grade 6 or above	-0.347	-0.598**	1.511	-0.455	-2.373
	(0.221)	(0.266)	(1.355)	(0.595)	(2.228)
Zone fixed effects	√	√	√	√	√
N	408	408	274	111	80
Num. of dep. variables (= 1)	111	274	-	80	-
R-squared	0.068	0.049	0.100	0.110	0.173
p-value of the listed variables	0.003	0.029	0.014	0.363	0.081

The table reports OLS estimates where the unit of observation is the killing site. Out of 433 killing sites in columns 1-2 and 115 killing sites in column 4, 25 and 4 sites, respectively, are excluded because of a lack of information about the educational levels of non-migrant women aged 36-50 around the sites; 274 and 80 killing sites with information about victims are analyzed in columns 3 and 5, respectively. The dependent variable in column 1 is an indicator variable equal to 1 if killing sites have balanced spatial clusters and 0 otherwise. The dependent variable in columns 2 and 4 is an indicator variable equal to 1 if killing sites have information about victims and 0 otherwise. The dependent variable in columns 3 and 5 is the logarithmic value of the lower bound of the number of victims at each killing site. The education levels of non-migrant women aged 36-50 are the ones of non-migrant women aged 36-50 living in villages within 3.0 km of each killing site. Robust standard errors are reported in parentheses. p-values are from F-tests for the joint significance of the three listed variables. *** $p < 0.01$; ** $p < 0.05$; and * $p < 0.1$.

Table M: Impacts of genocide on children's educational outcomes

Subsample:	1977-79	1980	1981-82	1977-79	1980	1981-82
	[1]	[2]	[3]	[4]	[5]	[6]
Cohort:	Children aged 15-21			Children aged 6-14		
Dependent variable:	Years of schooling			Grade progression		
	A. Global Sample					
Genocidal Violence I	0.136**	0.119*	0.269***	-0.008	0.000	0.057**
	(0.062)	(0.068)	(0.056)	(0.035)	(0.036)	(0.028)
Mean (≥ 3.0 km)	4.038	4.218	4.075	-3.712	-3.656	-3.637
N	11,141	10,642	19,271	10,520	10,185	18,661
N (< 3.0 km of K.S.)	2,886	2,602	4,654	2,697	2,490	4,476
R-squared	0.357	0.325	0.299	0.150	0.155	0.160
	B. Local Sample I (all spatial clusters)					
Genocidal Violence I	0.029	-0.013	0.192***	-0.041	0.000	-0.011
	(0.085)	(0.089)	(0.070)	(0.049)	(0.047)	(0.034)
Mean (≥ 3.0 km)	4.343	4.527	4.403	-3.588	-3.554	-3.488
N	5,738	5,474	9,744	5,402	5,246	9,416
N (< 3.0 km of K.S.)	2,632	2,398	4,316	2,460	2,294	4,149
R-squared	0.395	0.360	0.327	0.218	0.209	0.208
	C. Local Sample II (balanced spatial clusters)					
Genocidal Violence I	-0.355**	-0.191	-0.018	-0.148*	-0.003	0.027
	(0.146)	(0.158)	(0.118)	(0.088)	(0.073)	(0.054)
Mean (≥ 3.0 km)	4.515	4.61	4.530	-3.547	-3.565	-3.441
N	2,137	2,154	4,011	2,027	2,068	3,900
N (< 3.0 km of K.S.)	965	945	1,764	908	909	1,705
R-squared	0.454	0.401	0.350	0.283	0.265	0.236
	D. Local Sample III (all spatial clusters)					
Genocidal Violence II	-0.010	0.077	0.115	-0.061	0.030	0.026
	(0.090)	(0.096)	(0.078)	(0.053)	(0.054)	(0.038)
Mean of the outcome (< 6.0 km)	4.299	4.278	4.268	-3.617	-3.601	-3.555
Mean of Genocidal Violence II	6.020	5.915	5.802	6.018	5.927	5.810
S.D. of Genocidal Violence II	1.920	1.845	1.867	1.917	1.844	1.864
N	2,581	2,349	4,171	2,418	2,250	4,036
R-squared	0.409	0.363	0.330	0.220	0.217	0.215
	E. Local Sample IV (balanced spatial clusters)					
Genocidal Violence II	-0.372***	0.035	0.026	-0.186**	-0.006	0.054
	(0.126)	(0.143)	(0.113)	(0.081)	(0.072)	(0.052)
Mean of the outcome (< 6.0 km)	4.176	4.177	4.171	-3.669	-3.623	-3.548
Mean of Genocidal Violence II	6.022	5.864	5.651	6.018	5.864	5.647
S.D. of Genocidal Violence II	1.736	1.742	1.707	1.740	1.749	1.710
N	1,008	1,015	1,798	951	974	1,752
R-squared	0.440	0.362	0.335	0.234	0.232	0.229

The table reports OLS estimates where the unit of observation is the household. Robust standard errors, adjusted for clustering by village, are reported in parentheses. Global Sample − households in the districts surveyed by DC-Cam; Local Sample I − households within 6.0 km of killing sites; Local Sample II − households within 6.0 km of the selected killing sites (6.0 km balanced spatial clusters); Local Sample III − households within 6.0 km of killing sites with victim information; Local Sample IV − households within 6.0 km of the selected killing sites with victim information (6.0 km balanced spatial clusters). "Years of schooling" is the average years of schooling of children aged 15-21. "Grade progression" is the average grade progression of children aged 6-14 measured by Grade - (Age - 5). See the text for the definitions of "Genocidal Violence I" and "Genocidal Violence II." In all regressions, the following variables are controlled: mother's age, father's age, a set of dummy variables for mothers' and fathers' educational attainment (grade 1-5 and grade 6 or above), three variables on village characteristics (the distance to major roads (km)), the proportion of non-migrant women aged 36-50 with grade 1-5, and the proportion of non-migrant women aged 36-50 with grade 6 or above), zone and district fixed effects, and spatial cluster fixed effects (only in panels B, C, D, and E). *** $p < 0.01$; ** $p < 0.05$; and * $p < 0.1$.

Table N: Robustness check – alternative size of spatial clusters (4.0 km)

Subsample:	1977-79	1980	1981-82	1977-79	1980	1981-82
	[1]	[2]	[3]	[4]	[5]	[6]
Cohort:	Children aged 15-21			Children aged 6-14		
Dependent variable:	Years of schooling			Grade progression		
	A. Local Sample II					
Genocidal Violence I	-0.055<br>(0.133)	-0.103<br>(0.153)	0.015<br>(0.117)	-0.006<br>(0.083)	0.023<br>(0.073)	0.058<br>(0.061)
Mean ($\geq$ 2.0 km)	4.691	4.900	4.835	-3.483	-3.473	-3.369
N	2,294	2,210	4,005	2,165	2,129	3,858
N ($<$ 2.0 km of K.S.)	1,009	976	1,707	947	933	1,634
	B. Local Sample IV					
Genocidal Violence II	-0.063<br>(0.095)	-0.043<br>(0.097)	-0.041<br>(0.108)	-0.033<br>(0.045)	0.033<br>(0.044)	0.044<br>(0.049)
Mean of the outcome ($<$ 4.0 km)	4.648	4.534	4.713	-3.455	-3.505	-3.372
Mean of Genocidal Violence II	6.149	5.903	5.786	6.153	5.894	5.785
S.D. of Genocidal Violence II	1.927	1.891	1.812	1.931	1.900	1.804
N	1,067	1,014	1,792	1,001	982	1,738

See the notes to Table M.

Table O: Robustness check – alternative size of spatial clusters (8.0 km)

Subsample:	1977-79	1980	1981-82	1977-79	1980	1981-82
	[1]	[2]	[3]	[4]	[5]	[6]
Cohort:	Children aged 15-21			Children aged 6-14		
Dependent variable:	Years of schooling			Grade progression		
	A. Local Sample II					
Genocidal Violence I	-0.094	0.428**	0.052	-0.038	-0.019	-0.008
	(0.167)	(0.171)	(0.129)	(0.098)	(0.091)	(0.067)
Mean ($\geq$ 4.0 km)	4.084	4.179	4.128	-3.739	-3.691	-3.625
N	1,914	1,914	3,511	1,827	1,830	3,407
N (< 4.0 km of K.S.)	969	968	1,839	929	926	1,785
	B. Local Sample IV					
Genocidal Violence II	-0.013	-0.014	0.025	-0.077	-0.034	-0.008
	(0.137)	(0.108)	(0.118)	(0.084)	(0.055)	(0.043)
Mean of the outcome (< 8.0 km)	3.958	4.020	3.908	-3.759	-3.731	-3.682
Mean of Genocidal Violence II	6.047	5.934	5.712	6.042	5.941	5.708
S.D. of Genocidal Violence II	1.715	1.736	1.748	1.716	1.740	1.747
N	924	888	1,541	874	848	1,500

See the notes to Table M.

Table P: Robustness check – alternative continuous genocide measures

Subsample:	1977-79	1980	1981-82	1977-79	1980	1981-82
	[1]	[2]	[3]	[4]	[5]	[6]
Cohort:	Children aged 15-21			Children aged 6-14		
Dependent variable:	Years of schooling			Grade progression		
	A. Quadratic polynomial in distance					
Genocidal Violence II	-0.215***	0.003	0.018	-0.110**	-0.010	0.033
	(0.070)	(0.074)	(0.064)	(0.045)	(0.040)	(0.028)
Mean of the outcome (< 6.0 km)	4.176	4.177	4.171	-3.669	-3.623	-3.548
Mean of Genocidal Violence II	4.794	4.677	4.412	4.786	4.678	4.408
S.D. of Genocidal Violence II	2.037	2.093	1.987	2.047	2.107	1.994
N	1,008	1,015	1,798	951	974	1,752
R-squared	0.441	0.362	0.335	0.235	0.232	0.229
	B. Cubic polynomial in distance					
Genocidal Violence II	-0.143***	-0.002	0.014	-0.075**	-0.009	0.023
	(0.048)	(0.049)	(0.044)	(0.030)	(0.027)	(0.019)
Mean of the outcome (< 6.0 km)	4.176	4.177	4.171	-3.669	-3.623	-3.548
Mean of Genocidal Violence II	3.581	3.506	3.185	3.568	3.508	3.182
S.D. of Genocidal Violence II	2.445	2.561	2.377	2.460	2.583	2.389
N	1,008	1,015	1,798	951	974	1,752
R-squared	0.441	0.362	0.335	0.235	0.232	0.230

See the notes to Table M.

Table Q: Sensitivity analysis – Local Sample II (binary genocide measure)

Subsample:	1977-79	1980	1981-82	1977-79	1980	1981-82
	[1]	[2]	[3]	[4]	[5]	[6]
Cohort:	Children aged 15-21			Children aged 6-14		
Dependent variable:	Years of schooling			Grade progression		
	Local Sample II (balanced spatial clusters): Genocidal Violence I					
Baseline model (age, education, village characteristics, regional fixed effects)	-0.355** (0.146)	-0.191 (0.158)	-0.018 (0.118)	-0.148* (0.088)	-0.003 (0.073)	0.027 (0.054)
R-squared (R^2_{BM})	0.454	0.401	0.350	0.283	0.265	0.236
	A. Sensitivity to removals of covariates					
Restricted model I (age, education, regional fixed effects)	-0.334** (0.157)	-0.144 (0.171)	0.069 (0.131)	-0.133 (0.086)	0.005 (0.076)	0.050 (0.055)
R-squared	0.426	0.373	0.323	0.277	0.260	0.226
Restricted model II (age, village characteristics, regional fixed effects)	-0.306** (0.155)	-0.165 (0.166)	-0.029 (0.124)	-0.130 (0.090)	0.007 (0.073)	0.022 (0.055)
R-squared	0.376	0.336	0.290	0.250	0.238	0.203
Restricted model III (age, regional fixed effects)	-0.254 (0.180)	-0.082 (0.194)	0.110 (0.153)	-0.104 (0.089)	0.025 (0.078)	0.061 (0.060)
R-squared	0.305	0.272	0.227	0.232	0.223	0.177
Restricted model IV (regional fixed effects)	-0.260 (0.180)	-0.075 (0.194)	0.112 (0.153)	-0.105 (0.089)	0.025 (0.078)	0.065 (0.060)
R-squared ($R^2_{RM_{IV}}$)	0.304	0.271	0.225	0.232	0.221	0.176
	B. Coefficient bounds (γ^*)					
	$R^2_{max} = 1.5 \times R^2_{BM}$					
$\delta = 1$	-0.498	-0.370	-0.200	-0.269	-0.086	-0.047
$\delta = -1$	-0.212	-0.013	0.164	-0.028	0.081	0.101
	$R^2_{max} = 2.0 \times R^2_{BM}$					
$\delta = 1$	-0.642	-0.548	-0.381	-0.389	-0.170	-0.121
$\delta = -1$	-0.069	0.165	0.346	0.092	0.164	0.175
	$R^2_{max} = 1$					
$\delta = 1$	-0.700	-0.725	-0.693	-0.757	-0.468	-0.453
$\delta = -1$	-0.011	0.342	0.658	0.461	0.462	0.507

The table reports OLS estimates where the unit of observation is the household. Robust standard errors, adjusted for clustering by village, are reported in parentheses. See the notes to Table M for the sample, the dependent variables, and the genocide measures. Panel A assesses the sensitivity of the results based on the baseline mode to removing observed covariates. In Baseline model, the following covariates are controlled: age (mother's age, father's age), education (a set of dummy variables for mother's and father's educational attainment (grade 1-5 and grade 6 or above)), village characteristics (the distance to major roads (km)), the proportion of non-migrant women aged 36-50 with grade 1-5, and the proportion of non-migrant women aged 36-50 with grade 6 or above), and regional fixed effects (zone and district fixed effects). Spatial cluster fixed effects are always controlled for. In Restricted models I-IV, the following covariates are controlled: Restricted model I – age, education, and regional fixed effects; Restricted model II – age, village characteristics, regional fixed effects; Restricted model III – age, regional fixed effects; Restricted model IV – regional fixed effects. Panel B considers the coefficient bounds based on Oster's approach. See Section 5 for detailed explanation. *** $p < 0.01$; ** $p < 0.05$; and * $p < 0.1$.

Table R: Sensitivity analysis – Local Sample IV (continuous genocide measure)

Subsample:	1977-79	1980	1981-82	1977-79	1980	1981-82
	[1]	[2]	[3]	[4]	[5]	[6]
Cohort:	Children aged 15-21			Children aged 6-14		
Dependent variable:	Years of schooling			Grade progression		
	Local Sample IV (balanced spatial clusters): Genocidal Violence II					
Baseline model (R^2_{BM})	-0.372***	0.035	0.026	-0.185**	-0.006	0.054
(age, education, village characteristics, regional fixed effects)	(0.126)	(0.143)	(0.113)	(0.081)	(0.072)	(0.052)
R-squared	0.440	0.362	0.335	0.234	0.232	0.229
	A. Sensitivity to removals of covariates					
Restricted model I	-0.322**	0.034	0.090	-0.175**	-0.006	0.071
(age, education, regional fixed effects)	(0.141)	(0.144)	(0.127)	(0.078)	(0.073)	(0.053)
R-squared	0.398	0.331	0.303	0.227	0.933	0.218
Restricted model II	-0.351***	-0.001	-0.014	-0.179**	-0.020	0.040
(age, village characteristics, regional fixed effects)	(0.128)	(0.158)	(0.114)	(0.082)	(0.072)	(0.053)
R-squared	0.349	0.306	0.274	0.203	0.201	0.198
Restricted model III	-0.276*	-0.019	0.063	-0.159**	-0.025	0.062
(age, regional fixed effects)	(0.157)	(0.171)	(0.141)	(0.079)	(0.074)	(0.058)
R-squared	0.253	0.240	0.200	0.184	0.184	0.169
Restricted model IV	-0.273*	-0.027	0.063	-0.150*	-0.027	0.063
(regional fixed effects)	(0.159)	(0.172)	(0.141)	(0.079)	(0.073)	(0.057)
R-squared ($R^2_{RM_{IV}}$)	0.252	0.237	0.199	0.182	0.180	0.167
	B. Coefficient bounds (γ^*)					
	$R^2_{max} = 1.5 \times R^2_{BM}$					
$\delta = 1$	-0.487	0.123	-0.018	-0.266	0.040	0.036
$\delta = -1$	-0.257	-0.054	0.071	-0.105	-0.052	0.071
	$R^2_{max} = 2.0 \times R^2_{BM}$					
$\delta = 1$	-0.603	0.212	-0.063	-0.347	0.086	0.019
$\delta = -1$	-0.141	-0.142	0.116	-0.024	-0.098	0.089
	$R^2_{max} = 1$					
$\delta = 1$	-0.665	0.346	-0.152	-0.712	0.298	-0.064
$\delta = -1$	-0.079	-0.277	0.204	0.341	-0.309	0.172

See the notes to Table Q.
