# Supplementary material for: GIS for empirical research design: An illustration with georeferenced point data
Source: PLoS One. 2019 Mar 4;14(3):e0212316. doi: 10.1371/journal.pone.0212316 (PMC6398843; doi:10.1371/journal.pone.0212316)
Supplement: S1 File — This zip file contains the dataset and Stata do-file to replicate the empirical analyses presented in the case study. (ZIP) [file pone.0212316.s009.zip › S1_File/ReadMe.pdf]

GIS for Empirical Research Design: An Illustration with Georeferenced Point Data
S1 File – ReadMe

Katsuo Kogure (Kyoto University)
Yoshito Takasaki (University of Tokyo)

S1 File consists of three folders: (1) Data, (2) Figures, and (3) Tables.

(1) Data
The folder contains thirteen Stata-format datasets.

study_population.dta
Study population dataset – 43,535 households residing in districts surveyed and not surveyed by DC-Cam

global_sample.dta
Global sample dataset – 41,054 households residing in districts surveyed by DC-Cam

local_sample1.dta
Local sample I dataset – 20,956 households residing in villages within 6.0 km of killing sites

local_sample2.dta
Local sample II dataset – 8,302 households residing in villages within 6.0 km of selected killing sites (6.0 km balanced spatial clusters)

local_sample3.dta
Local sample III dataset – 9,105 households residing in villages within 6.0 km of killing sites with complete victim information

local_sample4.dta
Local sample IV dataset – 3,821 households residing in villages within 6.0 km of selected killing sites (6.0 km balanced spatial clusters) with complete victim information

local_sample2_4km.dta

Alternative Local sample II dataset – 8,509 households residing in villages within 4.0 km of selected killing sites (4.0 km balanced spatial clusters)

local_sample2_8km.dta
Alternative Local sample II dataset – 7,339 households residing in villages within 8.0 km of selected killing sites (8.0 km balanced spatial clusters)

local_sample4_4km.dta
Alternative Local sample IV dataset – 3,873 households residing in villages within 4.0 km of selected killing sites (4.0 km balanced spatial clusters) with complete victim information

local_sample4_8km.dta
Alternative Local sample IV dataset – 3,353 households residing in villages within 8.0 km of selected killing sites (8.0 km balanced spatial clusters) with complete victim information

killing_sites.dta
The dataset including killing-site characteristics

villages.dta
The dataset including village characteristics

spatial_cluster_6.0km_ks474.dta
The dataset contains the information of killing site 474 used in Table 2.

(2) Figures
The folder contains eight Stata do-files: Figs 3 and 4, and S1, S2, S4, S5, S6, and S7 Figs. Each do-file replicates the results of the corresponding figure using data stored in the Data folder.

(3) Tables
The folder contains eighteen Stata do-files: Tables 1, 2, A, B, D, E, F, G, H, I, J, L, M, N, O, P, Q, and R. Each do-file replicates the results of the corresponding table using data stored in the Data folder.
